# Tanshinol ameliorates imiquimod-induced psoriasis by inhibiting M1 macrophage polarization through suppression of the notch signaling pathway

**DOI:** 10.1007/s00210-024-03166-9

**Published:** 2024-06-04

**Authors:** Junhao Liu, Shuangshuang Yong, Sisi Yin, Jinhong Feng, Caihua Lian, Jie Chen

**Affiliations:** 1 Department of Dermatology, Tongchuan distric people’s hospital of dazhou, Dazhou, China; 2 Department of Dermatology, Dachuan distric people’s hospital of dazhou, Dazhou, China; 3 Department of Medical Aesthetics, Affiliated Hospital of Chengdu University of Traditional Chinese Medicine, Chengdu, China; 4 Department of Dermatology, Chengdu Seventh People’s Hospital (Affiliated Cancer Hospital of Chengdu Medical College), Chengdu, China; 5 Department of Dermatology, Gulin Traditional Chinese Medicine Hospital, Luzhou, China; 6 Department of Dermatology, Gulin Traditional Chinese Medicine Hospital, No.56, Luhong Road, Jinlan Street, Gulin County, Luzhou, Luzhou City, Sichuan Province P. R. China

**Keywords:** Psoriasis, Tanshinol, Macrophages polarization, Notch signaling pathway

## Abstract

**Background:**

Psoriasis is a common immune-related chronic inflammatory skin disease, often accompanied by significant itching, and once diseased, the course of the disease lasts for most of the lifetime. Tanshinol (TAN) is an active ingredient of *Salvia miltiorrhiza*, which possesses pharmacological effects such as anti-inflammatory and antioxidant properties. However, the effects of TAN on psoriasis have not been widely reported. Therefore, the aim of this study was to investigate the therapeutic effects and mechanisms of TAN in psoriasis.

**Methods:**

An imiquimod (IMQ)-induced psoriasis mouse model was constructed and treated with different doses of TAN to observe the changes in skin lesion phenotype, macrophage polarization, inflammation and Notch signaling pathway in mice. Further removal of macrophages or inhibition or activation of Notch signaling pathway was performed to examine the changes in skin lesion phenotype, macrophage polarization, inflammation and Notch signaling pathway in mice. In addition, *in vitro* experiments verified that TAN regulates RAW264.7 macrophage polarization and cytokine secretion through the Notch pathway.

**Results:**

The results showed that TAN alleviated IMQ-induced skin lesions and pathological phenotypes in psoriasis mice and inhibited Notch signaling pathway and M1-type macrophage polarization. Moreover, macrophage clearance and Notch signaling pathway activation inhibited the effect of TAN on psoriasis. Further *in vitro* experiments showed that Notch agonists reversed the effects of TAN on macrophage polarization and inflammatory cytokines.

**Conclusions:**

Collectively, these findings suggest that TAN may exert a therapeutic effect on psoriasis by inhibiting the Notch signaling pathway and thus M1-type macrophage polarization.

**Supplementary Information:**

The online version contains supplementary material available at 10.1007/s00210-024-03166-9.

## Introduction

Psoriasis is a chronic, inflammatory, relapsing skin disease that affects approximately 3% of the world’s population and is presented by scaly skin lesions, characterized by itching, pain and bleeding (Griffiths et al. 2021; Petit and Cano 2021). Studies have demonstrated that macrophages play an important role in the pathologic process of psoriasis, e.g., animal models of psoriasis have revealed that macrophages are present in large numbers in psoriasis-injured skin and that these cells play a key role in the pathogenesis of psoriasis-like skin inflammation (Kamata and Tada 2022, Kamiya et al. 2019). Furthermore, macrophages are an important source of TNF-α in skin injury, which is known to be a very essential cytokine in the pathologic process of psoriasis (Tao et al. 2022). Therefore, modulation of macrophage activation may be an important target for ameliorating psoriasis.

Notch signaling is highly conserved during cell growth and development and can act on a wide range of cell types to regulate cell proliferation, development, differentiation, and apoptosis, etc. (Zhou et al. 2022). It is also an important signaling pathway in the immune- inflammatory response, and its activation is also associated with macrophage polarization (Zhou et al. 2021). Studies have revealed that activation of Notch signaling increases the expression of M1 macrophage-related genes and promotes macrophage polarization from M2 to M1. In contrast, macrophages with typical Notch signaling deficiencies usually exhibit an M2 phenotype (Chen W. et al. 2021; Ma et al. 2022). Activation of Notch signaling also prevents the differentiation of M2-type macrophages by binding to Delta-like 4, which regulates M2-specific gene expression and apoptosis (Lin et al. 2018). So, whether activation of Notch signaling also exists in psoriasis to regulate the polarization state of macrophages, which in turn participates in and exacerbates the development of psoriasis is unclear.

Tanshinol is an active ingredient extracted from the traditional Chinese medicine *Salvia miltiorrhiza*, which possesses a variety of biological activities such as anti-inflammatory, antioxidant, anti-arthritic properties, and anti-tumor (Lai et al. 2021; Wang and Zhang 2020). Studies have reported that tanshinol can produce antitumor effects on breast cancer by restoring the anticancer activity of NK cells (Yang C. et al. 2021). Tanshinol also inhibited the activation of the FoxO3a transcription factor and the expression of the target genes Gadd45a and catalase, while counteracting the expression of alkaline phosphatase and osteoprotegerin (Yang et al. 2013, 2023). In addition, tanshinol has been used to treat and prevent various diseases such as atherosclerosis and liver fibrosis (Chen C. et al. 2016; Wang R. et al. 2018). However, whether it plays a role in the treatment of psoriasis remains unclear, and its molecular mechanism remains to be elucidated.

Thus, in this study, we will explore the regulatory effects of tanshinol on skin lesions, macrophage polarization, and Notch signaling in psoriasis mice, and analyze whether tanshinol can inhibit macrophage M1 polarization by regulating Notch signaling and thus ameliorate psoriasis by its effects and mechanisms.

## Materials and methods

### Experimental design

Fifty-four 7 ~ 8-week-old SPF-grade male BALB/c mice, with a body mass of 20 ~ 25 g, were purchased from Chengdu Dossy Experiment Animal Co. Ltd, (No. SCXK (Chuan) 2019-031). They were used for the experiment after being given a standard feed, free water, and acclimatization feeding for 1w at room temperature. All studies were conducted following the recommendations of the Guide for the Care and Use of Laboratory Animals, and every effort was made to minimize animal suffering and reduce the number of animals used in the experiments.

Mice were randomly divided into control group, model group, Tanshinol low-dose group (TAN-L), Tanshinol medium-dose group (TAN-M), Tanshinol high-dose group (TAN-H), Macrophage scavenger group (Clophosome-A, Clo-A), Tanshinol high-dose + macrophage scavenger group (TAN-H + Clo-A), Notch inhibitor group (DAPT), Tanshinol high dose + Notch agonist group (TAN-H + Jagged1), 6 mice in each group. The mice were shaved on the same part of the back and the size was about 2 cm×3 cm.

**Fig. 1:**
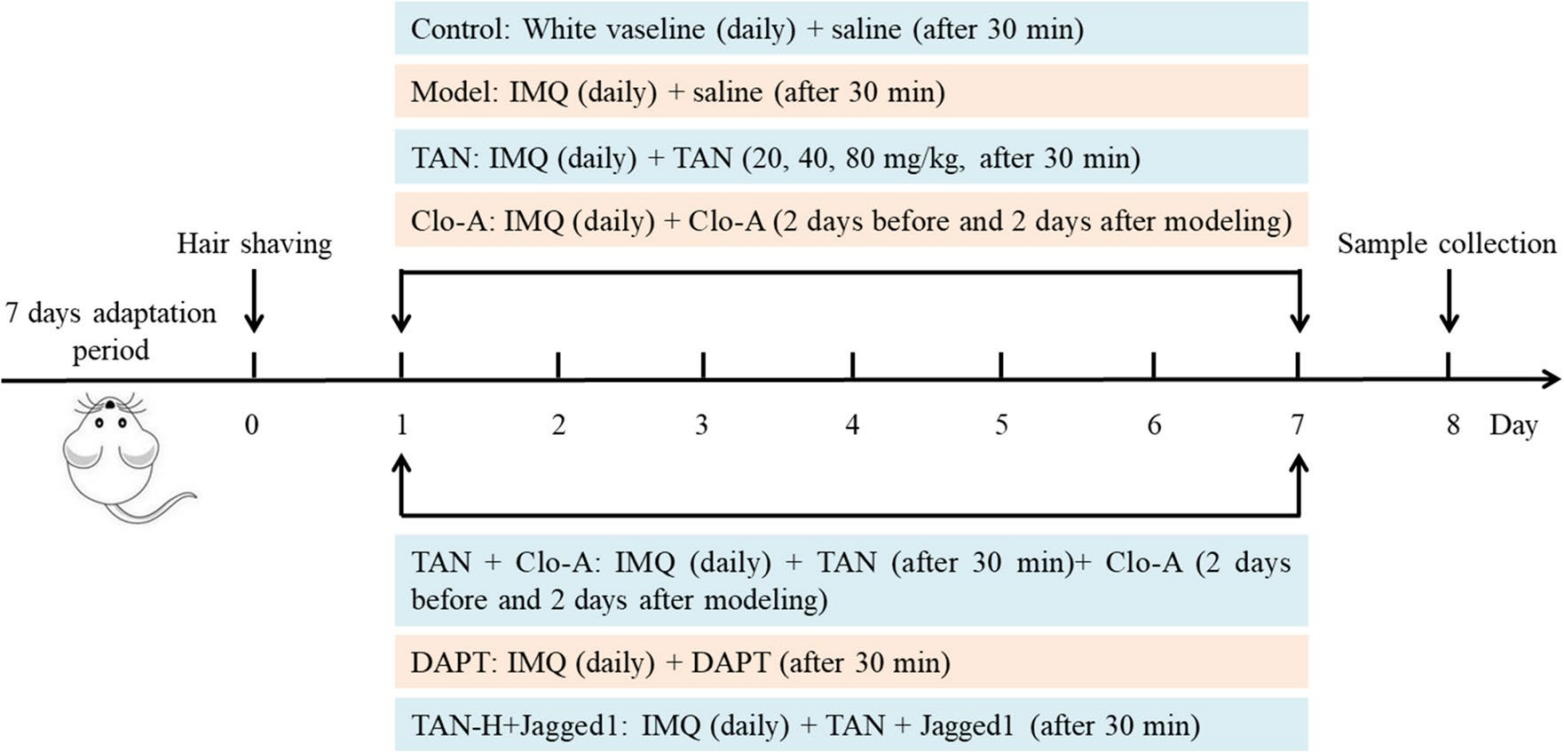
Illustration of experimental design. Day − 7 to Day − 1: mouse adaptation period. Day + 0: shaving. Day + 1 to Day + 7: imiquimod (IMQ) modeling + different treatments. Day + 8: killed, samples were collected for further measurements

Control group: 62.5 mg of white vaseline (Aladdin, Shanghai) was applied to the dorsal skin daily, and 100 µL of saline was injected intradermally after 30 min. Model group: 62.5 mg of 5% imiquimod cream (CAS: 99011-02-6, cat no: TF-6775, Molbase, Shanghai) was applied to the dorsal skin daily, and 100 µL of saline was injected intradermally after 30 min. TAN-L, TAN-M, and TAN-H groups: 62.5 mg of 5% imiquimod cream was applied to the dorsal skin daily, after 30 min, 0.5 mg/mL, 1 mg/mL, and 2 mg/mL of TAN saline solution (20 mg/kg, 40 mg/kg, 80 mg/kg, Pure-one Biotechnology, CAS: 22681-72-7, cat no: P0907, purity ≥ 98.0%, Shanghai) were gavaged respectively. Clo-A group: 62.5 mg of 5% imiquimod cream was applied to the dorsal skin daily, and Clo-A (1 mg/only, product no. F7010C-A, FormuMax Scientific Inc., USA) was injected intraperitoneally 2 days before and 2 days after modeling. TAN-H + Clo-A group: 62.5 mg of 5% imiquimod cream was applied to the dorsal skin daily, after 30 min, 2 mg/mL of TAN saline solution (80 mg/kg) was gavaged, and Clo-A (1 mg/only) was injected intraperitoneally 2 days before and 2 days after modeling. DAPT group: 62.5 mg of 5% imiquimod cream was applied to the dorsal skin daily, followed 30 min later by intraperitoneal injection of DAPT (50 mg/kg, cat no: D5942, Sigma-Aldrich, USA). TAN-H + Jagged1 group: 62.5 mg of 5% imiquimod cream was applied to the dorsal skin daily, after 30 min, 2 mg/mL of TAN saline solution (80 mg/kg) was gavaged, and intraperitoneal injection of Jagged1 (1 mg/kg, cat no: CF228277, ChemeGen, Shanghai), for 7 consecutive days in each group. Illustration of experimental design as shown in Fig. 1.

### General condition and ear thickness

High-resolution photographs were taken of the phenotype of mice, the degree of thinness and thickness, color changes, and relief of scales of dorsal skin lesions, and the degree of scales and redness in the ears. Measure the thickness of mice’s ears using vernier callipers at 3:00 p.m. on the 7 d, measure three times and take the average value.

### PASI score

The back skin erythema, scales, and infiltration in each mouse were PASI scored according to the following criteria. The PASI scores were 0 (none): no visible erythema or scales on the skin surface; 1 (light grade): part of the skin was covered by scales or dander with a light red color; 2 (moderate): majority of the skin was covered by sheeted scales and moderate ridges, and the edge of the plaque was red in color; 3 (severe): almost all skin was covered by thick and layered scales with obvious ridges, and the plaque was deep red; 4 (extremely severe): the whole skin was covered by especially thick and layered scales, extremely red in color was obvious ridges. The back skin erythema, scales and infiltration were scored together to obtain the total score.

### Hematoxylin-eosin (HE) staining

The dorsal skin lesion tissues of mice were taken, fixed with 4% paraformaldehyde for 24 h, paraffin tissue sections were performed with a thickness of 5 μm, HE staining was performed, and histopathological changes were observed using a fluorescence microscope (BA210Digital, Motic China Group Co., Ltd.).

### Immunofluorescence staining

Mouse skin lesion tissues were fixed by 4% paraformaldehyde, paraffin Sect. (3 μm) were prepared, microwave oven antigen repair in citrate buffer, endogenous peroxidase was blocked by 3% hydrogen peroxide, serum was blocked, and ki67 (1:400, cat no: HA721115, HUABIO, Beijing), K6 (1:100, cat no: ab93279, Abcam, UK), F4/80 (1:100, cat no: 28463-1-AP, Proteintech, Wuhan) primary antibody was incubated at 4℃ overnight, secondary antibody (FITC-labeled goat anti-rabbit, cat no: GB22303, Servicebio, Wuhan) was added and incubated at 37℃ for 30 min, DAPI was added dropwise and incubated at room temperature for 10 min, and images were captured using digital scanning and browsing software (OlyVIA, OLYMPUS, Japan) and the fluorescence intensity of all acquired images was measured using the Image-J analysis system (National Institutes of Health, USA).

### Immunofluorescence co-staining

The skin lesion tissues were fixed, paraffin-embedded, sliced sections (thickness 3 μm), and dewaxed. Then, microwave oven antigen repair in citrate buffer, endogenous peroxidase was blocked by 3% hydrogen peroxide, serum was blocked. Next, add F4/80 antibody (1:100, cat no: 28463-1-AP, Proteintech, Wuhan) at 4℃ overnight, dropwise add HRP-labeled secondary antibody (1:100, cat no: GB23303, Servicebio, Wuhan) and incubate at room temperature for 30 min, add FITC-Tyramide (1:500, cat no: G1222, Servicebio, Wuhan) and incubate at room temperature for 10 min. Sections were submerged into citrate buffer microwaved for 10 min, followed by the addition of antibodies against iNOS (1:100, cat no: GB11119, Servicebio, Wuhan), CD206 (1:100, cat no: GB113497, Servicebio, Wuhan), and Notch1 (1:100, cat no: 11976R, Bioss, Beijing) incubated overnight at 4℃, and dropwise addition of fluorescent secondary antibody (CY3 labeled goat anti-rabbit, cat no: GB21303, Servicebio, Wuhan) was incubated at 37℃ for 30 min. Images were captured using digital scanning and browsing software (OlyVIA, OLYMPUS, Japan) and the fluorescence intensity of all acquired images was measured using the Image-J analysis system (National Institutes of Health, USA).

### Immunohistochemical staining

Mouse skin lesion tissues were fixed with 4% paraformaldehyde and embedded in paraffin, and Sect. (5 μm) were prepared, microwave oven antigen repair in citrate buffer, endogenous peroxidase was blocked by 3% hydrogen peroxide, serum was blocked. The primary antibody was incubated with MCP-1 (1:100, cat no: ab30852, Abcam, UK) and MIF (1:200, cat no: bs-1044R, Bioss, Beijing) at 4℃ overnight, and then incubated with secondary antibody (1:100, cat no: GB23303, Servicebio, Wuhan) at 37℃. DAB was added for color development, and hematoxylin was added for 3 min, and the slices were sealed with neutral gum. Images of the sections were acquired using a microcamera system (BA400Digital, Motic, Shanghai), and the % DAB Positive Tissue per image was calculated using the Halo data analysis system (Halo 101-WL-HALO-1, Indica labs, USA).

### Quantitative reverse transcription-polymerase chain reaction (qRT-PCR)

Total RNA was extracted from the skin lesion tissues samples using an ultra-pure RNA extraction kit (cat no: 9767, Takara, Japan), and 5 µL of RNA was taken to detect the integrity of RNA. The residual genomic DNA in the RNA was digested with a DNase I kit (cat no: 2270 A, Takara, Japan) and reverse transcription was performed using a reverse transcription kit (cat no: RR037Q, Takara, Japan). Amplification was performed using TB Green ^TM^ Premix Ex Taq™ II (cat no: RR420Q, Takara, Japan). The sequences of the primers used are listed in Table 1. The relative expression of each target gene was quantified by the 2^−ΔΔCt^ method; β-actin was used as an internal control.

**Table 1:** PCR Primer

Primer	Forward sequences (5’-3’)	Reverse sequences (5’-3’)
iNOS	ATCTTGGAGCGAGTTGTGGATTGTC	TAGGTGAGGGCTTGGCTGAGTG
TNF-α	ATGTCTCAGCCTCTTCTCATTC	GCTTGTCACTCGAATTTTGAGA
IL-6	CTCCCAACAGACCTGTCTATAC	CCATTGCACAACTCTTTTCTCA
IL-20	TAGTGTGCAAGCTGAAGATACA	CCCTGTCCAGATAGAATCTCAC
MIF	GACCAGCTCATGACTTTTAGC	AATAGTTGATGTAGACCCGGTC
MCP-1	TTTTTGTCACCAAGCTCAAGAG	TTCTGATCTCATTTGGTTCCGA
IL-4	TACCAGGAGCCATATCCACGGATG	TGTGGTGTTCTTCGTTGCTGTGAG
β-actin	CTACCTCATGAAGATCCTGACC	CACAGCTTCTCTTTGATGTCAC

### Macrophage culture and polarization

Mouse macrophage cell line RAW264.7 (ATCC, cat no: TIB-71, Manassas, VA) was inoculated into 96-well plates according to 2 × 10^4^ cells/mL and cultured with RPMI1640 culture medium containing 10% fetal bovine serum for 24 h. Cells were grouped into M0 control group (Control), M1 group, and M1 + TAN different concentration groups (2.5, 5, 10, 20, and 40 µmol/L). The Control group continued incubation for 24 h with RPMI1640 culture medium (containing 10% fetal bovine serum) containing an equal volume ratio of Dimethyl sulfoxide (DMSO, cat no: 67-68-5, Sigma, Shanghai). M1 group was co-stimulated with bacterial lipopolysaccharide (LPS, cat no: HY-D1056, MedChemExpress, USA) + 20 g/mL IFN-γ (cat no: I0320301, Sino Biological Inc., Beijing) for 24 h. M1 + TAN different concentration groups were treated with TAN (2.5, 5, 10, 20, and 40 µmol/L) with 100 ng/mL LPS + 20 ng/mL IFN-γ for 24 h. CCK-8 was used to screen the optimal intervention concentration of TAN. Subsequently, subgroups were set up: M0 control group (Control), M1 group, M1 + TAN-L group, M1 + TAN-M group, M1 + TAN-H group, M1 + Jagged1 (1 µM, cat no: CF228277, ChemeGen, Shanghai) group, the M1 + TAN-H + Jagged1 group. The cells were acted according to the grouping for 24 h.

### CCK-8 assay

The cells were collected and centrifuged at 3000 r/min for 5 min to obtain cell precipitates. The serum-free medium was diluted 1:10 with CCK-8 reagent (cat no: BS350B, Biosharp, Beijing), 110 µL/well of diluted CCK-8 working solution was added, and incubation was continued at 37℃ with 5% CO_2_ for 2 h. The optical density (OD) values of each well were measured at 450 nm using a spectra max PLUS 384 microplate reader (Molecular Devices, Shanghai).

### Enzyme-linked immunosorbent assay (ELISA)

The concentrations of inducible nitric oxide (iNOS, cat no: ZC-38,979), interleukin-6 (IL-6, cat no: ZC-37,988), interleukin-20 (IL-20, cat no: ZC-37,977), macrophage migration inhibitory factor (MIF, cat no: ZC-38,359), monocyte chemoattractant protein 1 (MCP-1, cat no: ZC-38,075), and interleukin-4 (IL-4, cat no: ZC-37,986) in the cells were measured with ELISA kits (Shanghai ZCIBIO Technology Co., Ltd), and the experimental procedures were strictly by the manufacturer’s instructions.

### Flow cytometry assay

Cells were collected, centrifuged at 3000 r/min for 5 min to obtain cell precipitates, resuspended with 100 µL PBS, and labeled with anti-human F4/80-PC7 (cat no: 123,126), CD86-APC (cat no: 200,316), CD163-PE (cat no: 111,803, Biolegend, USA) according to the instructions, and expression of these molecules was detected by FC500 flow cytometer (Beckman Coulter, USA), the expression levels were expressed as a percentage of positive cells.

### Western blotting

RIPA lysate (cat no: P0013, Beyotime, Shanghai) lysed cells and skin lesion tissues on ice to extract proteins, and BCA protein concentration assay kit (cat no: P0009, Beyotime, Shanghai) to determine protein concentration. Total proteins were electrophoresed on SDS-PAGE polyacrylamide gels and transferred to PVDF membranes, closed with 5% skimmed milk, and then incubated overnight at 4℃ with primary antibodies [Notch1 (cat no: A16673), Hey1 (cat no: A16110), Hes1 (cat no: A0925), Abclonal, Wuhan], secondary antibodies Goat Anti-Rabbit IgG (H + L) HRP (cat no: S0001, Affbiotech, Suzhou) incubated at room temperature for 1 h, developed by ECL, and the bands were exposed with Fluorescence Image Analysis System Software V2.0 (Tanon, Shanghai), and the results were scanned by Gel-Pro analyzer4 software and expressed as the integrated optical density (IOD) of the target protein.

### Statistical analysis

All data are shown as mean ± standard deviation (SD). The SPSS 19.0 software (SPSS Inc., Chicago, USA) was used to analyze data and statistical differences between groups were determined by one way ANOVA. The LSD-*t* test was used for multiple two-by-two comparisons when the variances were equal, and Tamhane’s T2 test was used when the variances were not equal. *P* < 0.05 were considered statistically significant.

## Results

### Effect of TAN on the phenotype of skin lesions in mice with psoriasis

**Fig. 2:**
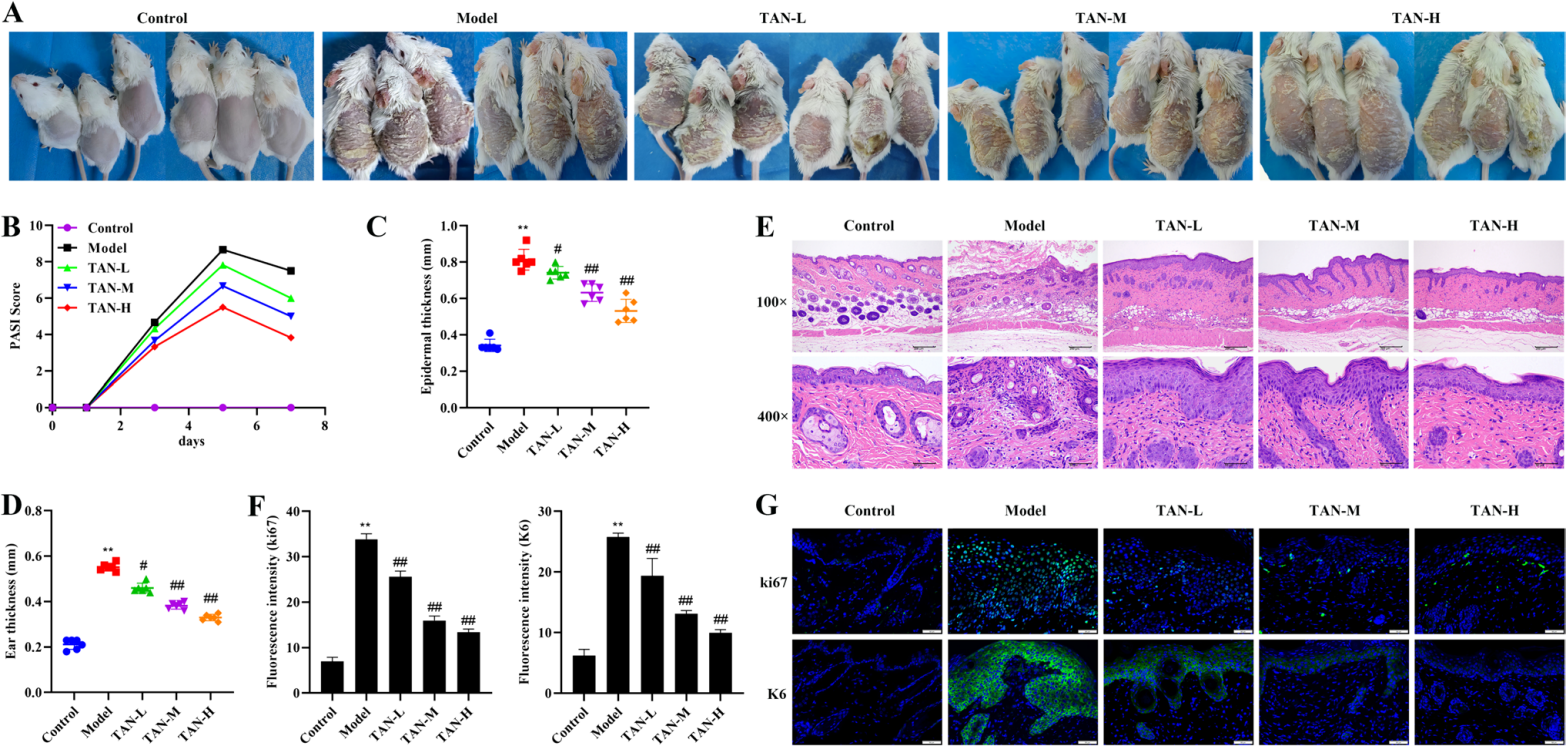
Effect of TAN on the phenotype of skin lesions in mice with psoriasis. **(A)** Photograph of the skin on the back of a mouse. **(B)** PASI score. **(C)** Epidermal thickness. **(D)** Ear thickness. **(E)** Histopathologic picture of the skin of the back (HE, Scale bar, 200 μm and 50 μm). **(F)** The expression of ki67 and K6 in dorsal skin tissues. **(G)** Immunofluorescence staining of ki67 and K6 (20×, Scale bar, 50 μm). Data were represented as mean ± SEM. Compared with control group, **P < 0.01; compared with model group, #*P* < 0.05, ##*P* < 0.01

First, we observed the improvement effect of TAN on the skin lesion phenotype of psoriasis mice. The clinical manifestations of the back skin of mice showed that the back skin of mice in the model group was thickened, with erythema, white scaly shoulders, and fissures in the back skin of some mice, and the PASI scores, epidermal thickness, and ear thickness increased significantly compared with those of the control group, which were significantly improved after treatment with different dosages of TAN (*P* < 0.05, Fig. 2A-D). Pathological tissues of skin lesions showed keratinization insufficiency, epidermal thickening, slight hemorrhage and a small amount of erythrocyte accumulation in mice of the model group, and TAN treatment attenuated the above pathological damage (Fig. 2E). In addition, the expression of ki67 and K6 was significantly elevated in the skin lesion tissues of the model group compared with the control group, and the expression of ki67 and K6 was significantly suppressed by the TAN-treated group compared with the model group (*P* < 0.05, Fig. 2F-G), suggesting that TAN could significantly alleviate the severity of psoriasis in mice.

### Effect of TAN on macrophage polarization and inflammation at skin lesion in psoriatic mice

**Fig. 3:**
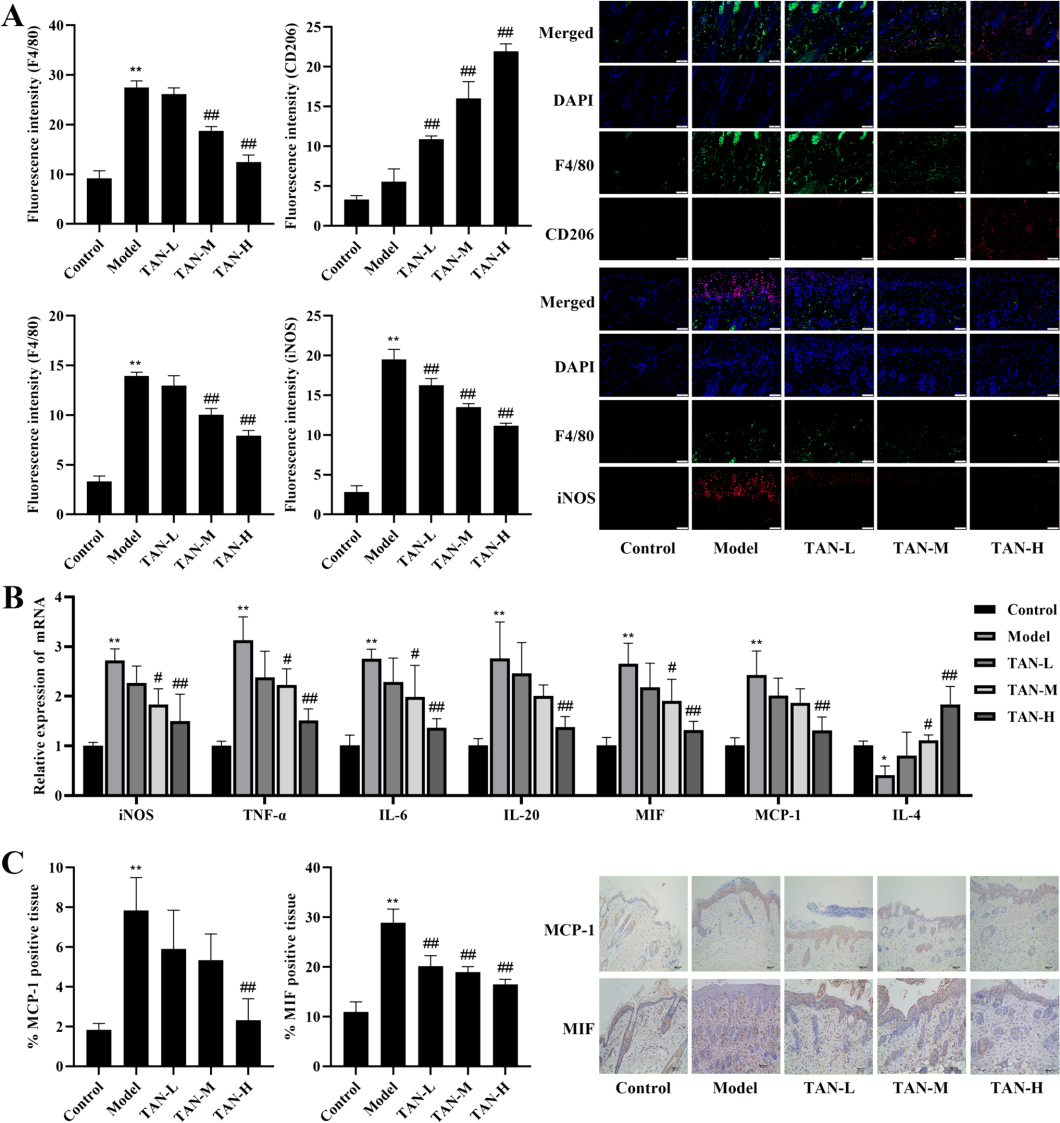
Effect of TAN on macrophage polarization and inflammation at skin lesion in psoriatic mice. **(A)** Immunofluorescence co-staining of F4/80 + iNOS and F4/80 + CD206 (20×, Scale bar, 50 μm). **(B)** The mRNA expression of iNOS, TNFα, IL-6, IL-20, MIF, MCP-1, IL-4 in dorsal skin tissues. **(C)** The expression of MCP-1 and MIF in dorsal skin tissues (immunohistochemical staining, 20×, Scale bar, 50 μm). Data were represented as mean ± SEM. Compared with control group, *P < 0.05, **P < 0.01; compared with model group, #*P* < 0.05, ##*P* < 0.01

Next, we examined the effect of TAN on macrophage polarization and inflammation at skin lesion in psoriatic mice. We found that the levels of iNOS (*P* < 0.05, Fig. 3A), mRNA expression of iNOS, TNFα, IL-6, IL-20, MIF, MCP-1 (*P* < 0.01, Fig. 3B), and the expression of MCP-1 and MIF (*P* < 0.01, Fig. 3C) were significantly increased in the dorsal skin tissues of the model group compared to the control group, while mRNA expression of IL-4 was significantly decreased (*P* < 0.05, Fig. 3B). Compared with the model group, the TAN-H group significantly reduced the levels of iNOS, TNFα, IL-6, IL-20, MIF, MCP-1, MIF, and significantly elevated the levels of CD206 and IL-4 (*P* < 0.01, Fig. 3), demonstrating the ameliorative effect of TAN-H on macrophage polarization and inflammation at the skin lesion site in psoriasis mice.

### Effect of macrophage clearance on TAN to ameliorate skin lesion phenotype in psoriasis mice

**Fig. 4:**
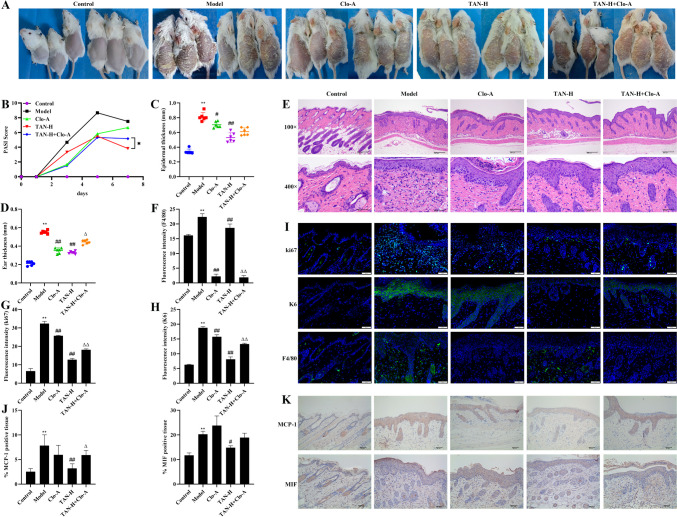
Effect of macrophage clearance on TAN to ameliorate skin lesion phenotype in psoriasis mice. **(A)** Photograph of the skin on the back of a mouse. **(B)** PASI score. **(C)** Epidermal thickness. **(D)** Ear thickness. **(E)** Histopathologic picture of the skin of the back (HE, Scale bar, 200 μm and 50 μm). **(F)** The expression of F4/80 in dorsal skin tissues. **(G)** The expression of ki67 in dorsal skin tissues. **(H)** The expression of K6 in dorsal skin tissues. **(I)** Immunofluorescence staining of ki67, K6, and F4/80 (20×, Scale bar, 50 μm). **(J-K)** The expression of MCP-1 and MIF in dorsal skin tissues (immunohistochemical staining, 20×, Scale bar, 50 μm). Data were represented as mean ± SEM. Compared with control group, **P < 0.01; compared with model group, #*P* < 0.05, ##*P* < 0.01; compared with the TAN-H group, Δ*P* < 0.05, ΔΔ*P* < 0.01

Since TAN affects psoriatic macrophage polarization, we cleared macrophages using Clo-A, and looked at the effect of TAN on the skin lesion phenotype in psoriatic mice. The results revealed that increased erythema, scaling and thickness of the skin on the back in the TAN-H + Clo-A group compared to the TAN-H group, as well as significantly higher PASI scores, and thickness of the ears (*P* < 0.05, Fig. 4A-D). Pathologic findings also showed increased epidermal thickness in the TAN-H + Clo-A group compared to the TAN-H group (Fig. 4E). The expression of ki67, K6, and MCP-1 was significantly increased and F4/80 was significantly reduced in the dorsal skin tissues of the TAN-H + Clo-A group compared with the TAN-H group (*P* < 0.05, Fig. 4F-K), showing that TAN may act on psoriasis through macrophages.

### Effect of TAN on notch signaling pathway in skin lesion tissues of psoriasis mice

**Fig. 5:**
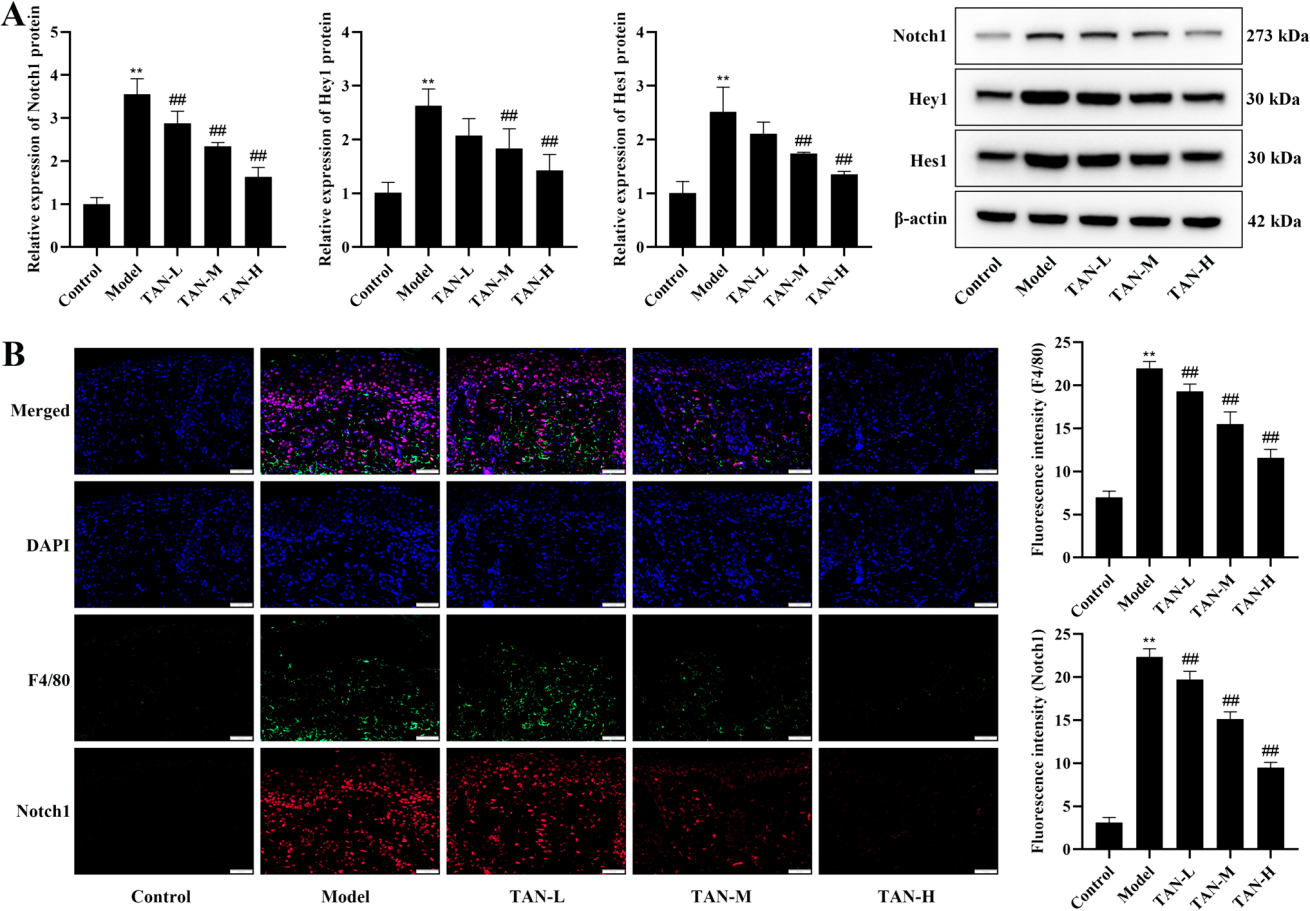
Effect of TAN on Notch signaling pathway in skin lesion tissues of psoriasis mice. **(A)** The protein expression of Notch1, Hey1, and Hes1 in dorsal skin tissues. **(B)** Immunofluorescence co-staining of F4/80 + Notch1 (20×, Scale bar, 50 μm). Data were represented as mean ± SEM. Compared with control group, **P < 0.01; compared with model group, ##*P* < 0.01

In this part, we analyzed the effect of TAN on Notch signaling pathway in skin lesion tissues of psoriasis mice, as shown in Fig. 5, the expression of Notch1, Hey1, and Hes1 in the dorsal skin tissues of the model group was significantly activated compared with that of the control group, and the TAN-M and TAN-H treatments significantly inhibited the expression of Notch1, Hey1, and Hes1 in the model mice (*P* < 0.01), revealing that TAN inhibited the Notch signaling pathway.

### Effect of TAN on skin lesion phenotype and macrophage polarization in psoriasis mice through inhibition of the Notch signaling pathway

**Fig. 6:**
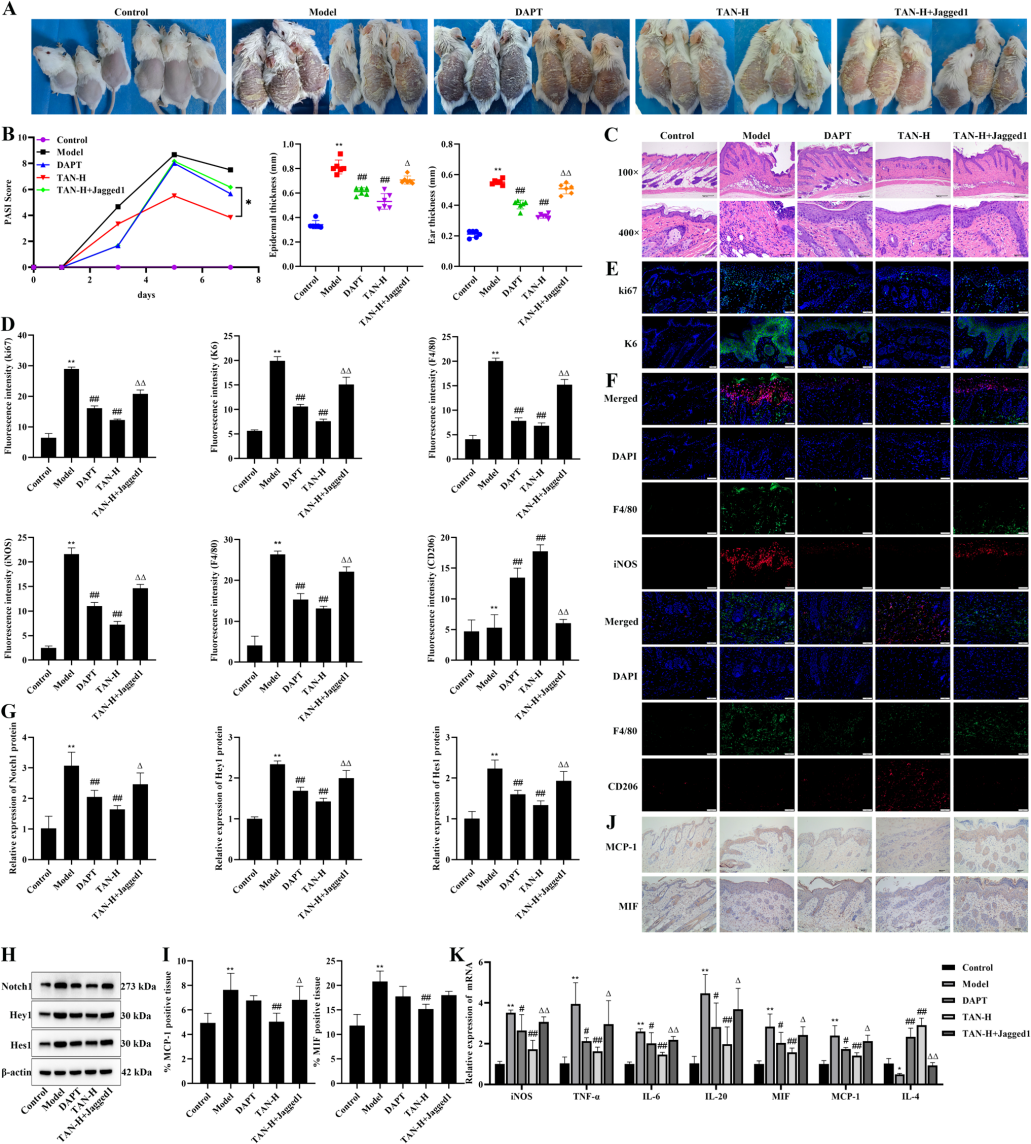
Effect of TAN on skin lesion phenotype and macrophage polarization in psoriasis mice through inhibition of the Notch signaling pathway. **(A)** Photograph of the skin on the back of a mouse. **(B)** PASI score, epidermal thickness, and ear thickness. **(C)** Histopathologic picture of the skin of the back (HE, Scale bar, 200 μm and 50 μm). **(D)** The expression of ki67, K6, F4/80 + iNOS, and F4/80 + CD206 in dorsal skin tissues. **(E)** Immunofluorescence staining of ki67 and K6 (20×, Scale bar, 50 μm). **(F)** Immunofluorescence co-staining of F4/80 + iNOS and F4/80 + CD206 (20×, Scale bar, 50 μm). **(G-H)** The protein expression of Notch1, Hey1, and Hes1 in dorsal skin tissues. **(I-J)** The expression of MCP-1 and MIF in dorsal skin tissues (immunohistochemical staining, 20×, Scale bar, 50 μm). **(K)** The mRNA expression of iNOS, TNFα, IL-6, IL-20, MIF, MCP-1, IL-4 in dorsal skin tissues. Data were represented as mean ± SEM. Compared with control group, *P < 0.05, **P < 0.01; compared with model group, #*P* < 0.05, ##*P* < 0.01; compared with the TAN-H group, Δ*P* < 0.05, ΔΔ*P* < 0.01

Further, we intervened in the Notch signaling pathway and analyzed the effects of TAN on skin lesion phenotype and macrophage polarization in psoriasis mice. The results showed that mice in the TAN-H + Jagged1 group had increased scaling and erythema on their backs and significantly higher PASI scores, epidermal thickness, ear thickness, and pathologic damage compared with the TAN-H group (*P* < 0.05, Fig. 6A-C). Compared with the TAN-H group, positive fluorescence staining for ki67, K6, iNOS was significantly increased, while CD206 was significantly decreased in the TAN-H + Jagged1 group (*P* < 0.05, Fig. 6D-F). Moreover, protein expression of Notch1, Hey1, and Hes1 was significantly promoted by TAN-H + Jagged1 (*P* < 0.05, Fig. 6G-H), and the expression of iNOS, TNFα, IL-6, IL-20, MIF, and MCP-1 was also significantly enhanced by TAN-H + Jagged1 (*P* < 0.05, Fig. 6I-K). Also, the Notch inhibitor DAPT acted on mice with similar effects as TAN-H, suggesting that Notch agonists reversed the therapeutic effects of TAN.

### *In vitro* experimental analysis of TAN regulation of macrophage polarization and cytokine secretion through the Notch pathway

**Fig. 7:**
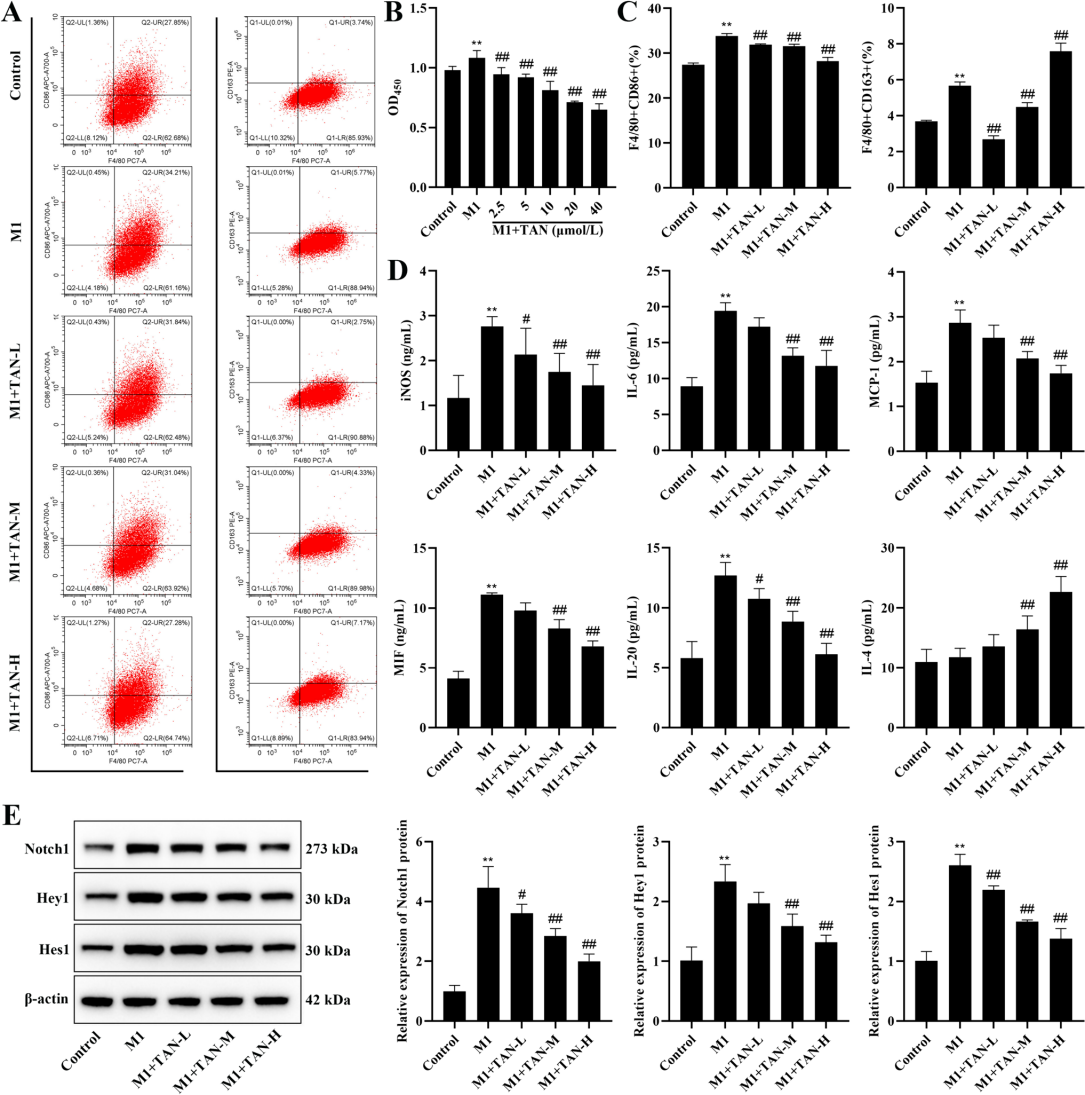
Effect of TAN on macrophage polarization, cytokine secretion and Notch pathway. **(A)** Flow cytogram of F4/80 + CD86 and F4/80 + CD163. **(B)** Cell viability. **(C)** Levels of F4/80 + CD86 and F4/80 + CD163. **(D)** Levels of iNOS, IL-6, IL-20, MIF, MCP-1, and IL-4. **(E)** The protein expression of Notch1, Hey1, and Hes1. Data were represented as mean ± SEM. Compared with control group, **P < 0.01; compared with M1 group, #*P* < 0.05, ##*P* < 0.01

Different concentrations of TAN intervened in macrophages, and cell viability was detected by CCK-8, and compared with the M1 group, the cell viability decreased with the increase of concentration (*P* < 0.01, Fig. 7B); therefore, 2.5 µmol/L, 10 µmol/L, and 40 µmol/L were chosen as the intervention concentrations of the TAN-L, TAN-M, and TAN-H groups, respectively, for the subsequent experiments. Next, we examined the levels of F4/80 + CD86 and F4/80 + CD163 in the cells, and the results showed that the F4/80 + CD86 + and F4/80 + CD163 level were significantly increased in M1 cells compared with the control (*P* < 0.01). TAN treatment significantly reduced the F4/80 + CD86 + levels and remarkably increased F4/80 + CD163 levels (*P* < 0.01, Fig. 7A, C). In addition, TAN treatment markedly decreased the levels of iNOS, IL-6, IL-20, MIF, MCP-1 and the protein expression of Notch1, Hey1, Hes1 and elevated the level of IL-4 in M1 cells (*P* < 0.05, Fig. 7D).

**Fig. 8:**
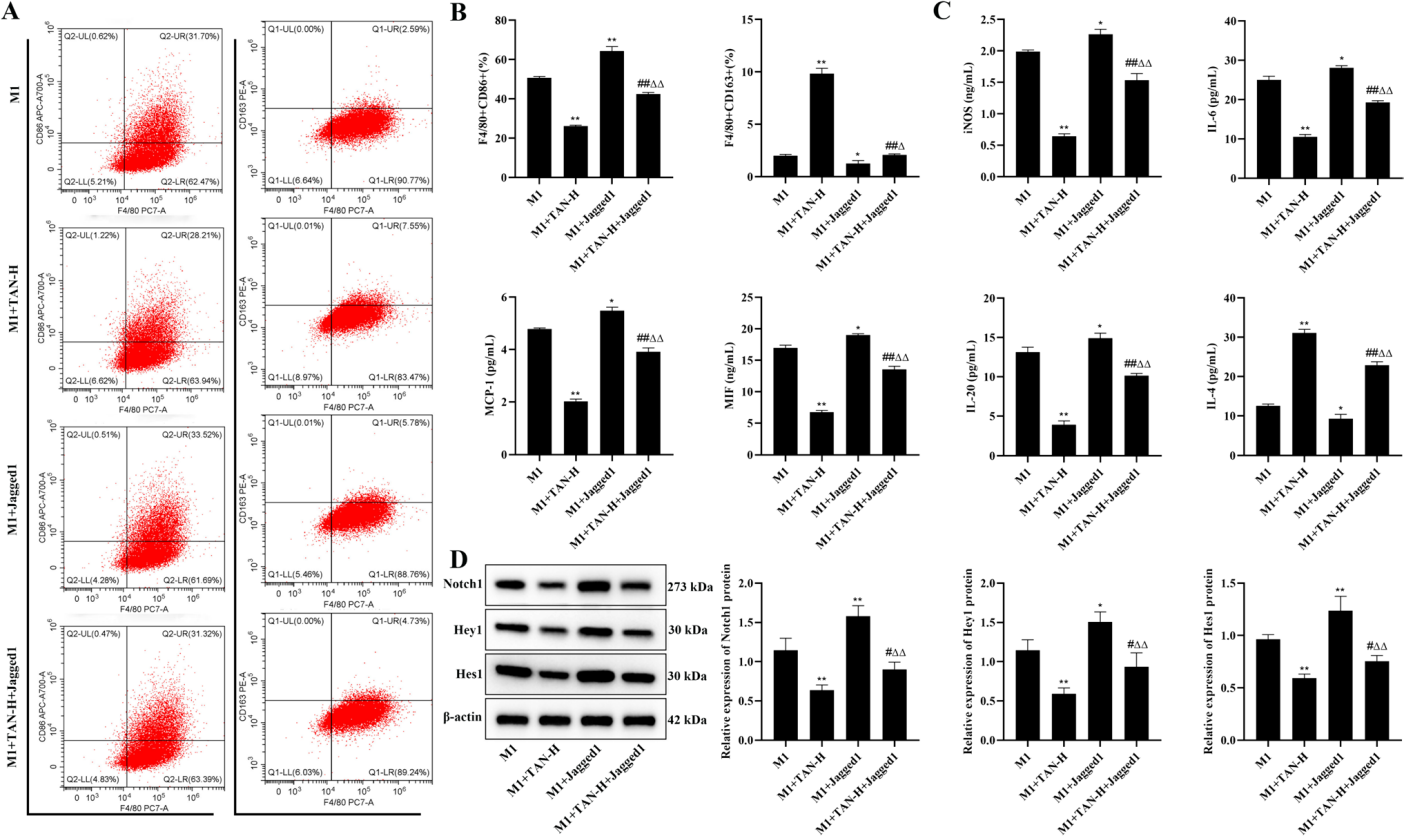
TAN regulates macrophage polarization and cytokine secretion through the Notch pathway. **(A)** Flow cytogram of F4/80 + CD86 and F4/80 + CD163. **(B)** Levels of F4/80 + CD86 and F4/80 + CD163. **(C)**. Levels of iNOS, IL-6, IL-20, MIF, MCP-1, and IL-4. **(D)** The protein expression of Notch1, Hey1, and Hes1. Data were represented as mean ± SEM. Compared with M1 group, *P < 0.05, **P < 0.01; compared with M1 + TAN-H group, #*P* < 0.05, ##*P* < 0.01; compared with M1 + Jagged1 group, Δ*P* < 0.05, ΔΔ*P* < 0.01

Also, the Notch agonist Jagged1 was used to intervene the cells, as shown in Fig. 8, compared with the M1 + TAN-H group, M1 + TAN-H + Jagged1 significantly increased the level of F4/80 + CD86, up-regulated the levels of iNOS, IL-6, IL-20, MIF, and MCP-1, and promoted Notch1, Hey1, and Hes1 protein expression (*P* < 0.05) and down-regulated the levels of F4/80 + CD163 and IL-4 (*P* < 0.05), demonstrating that Notch agonists reversed the effects of TAN on macrophage polarization and inflammatory cytokines.

## Discussions

In the present study, we found that TAN significantly alleviated the lesion-like phenotype of psoriasis, modulated macrophage polarization, inflammation, and inhibited the Notch signaling pathway. Moreover, macrophage clearance and Notch signaling pathway activation inhibited the effect of TAN on psoriasis. Further *in vitro* experiments showed that Notch agonists reversed the effects of TAN on macrophage polarization and inflammatory cytokines.

Currently, various natural mutant and transgenic animal models are being studied, but the most widely used is still imiquimod (IMQ)-induced mouse psoriasis-like model (Jabeen et al. 2020). IMQ is a Toll-like receptor (TLR7/8) agonist, which can induce inflammatory responses in mice, and its induced psoriasis-like model lesions in mice are characterized by increased epidermal hyperplasia, differentiation abnormalities, etc., and skin inflammation basically consistent with the pathological changes of psoriasis (Li et al. 2021; Silva-Abreu et al. 2023). In this study, we used IMQ to establish a mouse model of psoriasis. The results of the experiment showed that the skin thickness, scales and erythema on the back of the model mice increased, and the PASI score was up-regulated, indicating that the model mice were successfully established.

Hyperproliferation of epidermal keratinocytes and infiltration of dermal inflammatory factors are the most typical clinicopathologic features of psoriasis (Kelemen et al. 2022). Literature have suggested that stimulation by external factors can lead to abnormally high expression of keratins (K6, K16, K17) in keratinocytes, which are directly involved in regulating keratinocyte proliferation, migration, and inflammatory responses (Funakoshi et al. 2020). Therefore, keratins are considered to be important markers for evaluating the degree of proliferation in psoriasis (Chen et al. 2022a). In this study, we found that IMQ promotes the proliferation of keratinocytes and mouse epidermis, as evidenced by elevated expression of Ki67 and keratin K6, and that TAN has a role in inhibiting the most prominent pathological features of psoriasis. Additionally, keratinocytes have an intrinsic immune function, which can further enhance the inflammatory response in psoriasis by releasing a variety of inflammatory factors, such as causing the cells to secrete a variety of cytokines, chemokines, and antimicrobial peptides involved in regulating the immune response (Chen et al. 2022b). In this study, TAN significantly inhibited the levels of iNOS, TNFα, IL-6, IL-10, IL-20, MIF, and MCP-1 in psoriasis mice, which had a wide range of inflammatory inhibitory effects.

Studies have reported the presence of macrophage activation and aggregation in psoriatic lesions, which may induce hyperproliferation and aberrant differentiation of keratinocytes through the secretion of cytokines such as TNF-a, MIF, IL-6 and IL-20 (Tao et al. 2022). M1-type macrophages secrete inflammatory factors, such as IL-1β, IL-6, and IL-12, which are mainly pro-inflammatory (Schuster et al. 2020). M2-type macrophages secrete chemokines and matrix metalloproteinases, which are mainly anti-inflammatory (Wang Y. et al. 2023). Studies have demonstrated that shikonin combined with methotrexate modulates macrophage polarization, as evidenced by a decrease in the number of CD86 and an increase in the number of CD206, as well as changes in the levels of the M1-type markers iNOS, IL-1β, and TNF-α, and the M2-type markers Arg-1 and IL-10 (Tao et al. 2022). In the present study, similar to its reported results (Tao et al. 2022), we found that TAN inhibited the levels of M1-type markers iNOS, TNF-α and IL-6 and promoted the levels of M2-type markers CD206 and IL-4 in psoriatic mice, and that macrophage clearance inhibited the effects of TAN in psoriatic mice. In addition, TAN also modulated M1/M2 polarization and altered the levels of M1 and M2 markers in LPS-stimulated mouse macrophage RAW264.7 cells, which was consistent with the *in vivo* experiments. It is suggested that TAN promotes the polarization of psoriatic macrophages toward the anti-inflammatory phenotype M2 type.

Numerous studies have shown that Notch signaling is involved in and exacerbates a variety of autoimmune diseases by modulating macrophage polarization (Chen et al. 2021; Yan et al. 2024). For example, in autoimmune neuritis, blockade of the Notch signaling pathway effectively attenuates the extent of inflammatory infiltration (Ren et al. 2022). In an animal model of rheumatoid arthritis, Notch1 and its downstream target molecule Hes are highly expressed in macrophages, and the administration of Notch inhibitors reduces the inflammatory infiltration and the expression of inflammatory factors (Zhao et al. 2023). The use of Notch inhibitors was also found to reduce the inflammatory response in systemic lupus erythematosus (Breitkopf et al. 2020). In the present study, the Notch signaling pathway was activated in psoriatic mice, and TAN inhibited the expression levels of key Notch signaling pathway proteins. To further investigate the effect of Notch signaling pathway on macrophage polarization, we performed *in vivo* and *in vitro* experiments to overexpress or block Notch signaling pathway. The results revealed that DATP inhibited M1-type macrophage polarization, which was significantly increased by the addition of Jagged1, and the skin lesion-like phenotype of mice was aggravated by the addition of Jagged1. It is suggested that TAN may inhibit M1-type macrophage polarization by inhibiting the Notch signaling pathway.

Overall, we concluded that TAN alleviated IMQ-induced skin lesions and pathological phenotypes in psoriasis mice and inhibited Notch signaling pathway and M1-type macrophage polarization. In addition, TAN may exert a therapeutic effect on psoriasis by inhibiting the Notch signaling pathway and thus M1-type macrophage polarization.

## Electronic supplementary material

Below is the link to the electronic supplementary material.

Supplementary Material 1

Supplementary Material 2

Supplementary Material 3

## Data Availability

No datasets were generated or analysed during the current study.

## Abbreviations

TAN: Tanshinol
IMQ: imiquimod
