# Supplementary figures and images for: Tanshinol ameliorates imiquimod-induced psoriasis by inhibiting M1 macrophage polarization through suppression of the notch signaling pathway
Source: Naunyn Schmiedebergs Arch Pharmacol. 2024 Jun 4;397(11):8745–58. doi: 10.1007/s00210-024-03166-9 (PMC11522191; doi:10.1007/s00210-024-03166-9)

## Control

## M1

## M1+TAN-L

## M1+TAN-M

## M1+TAN-H

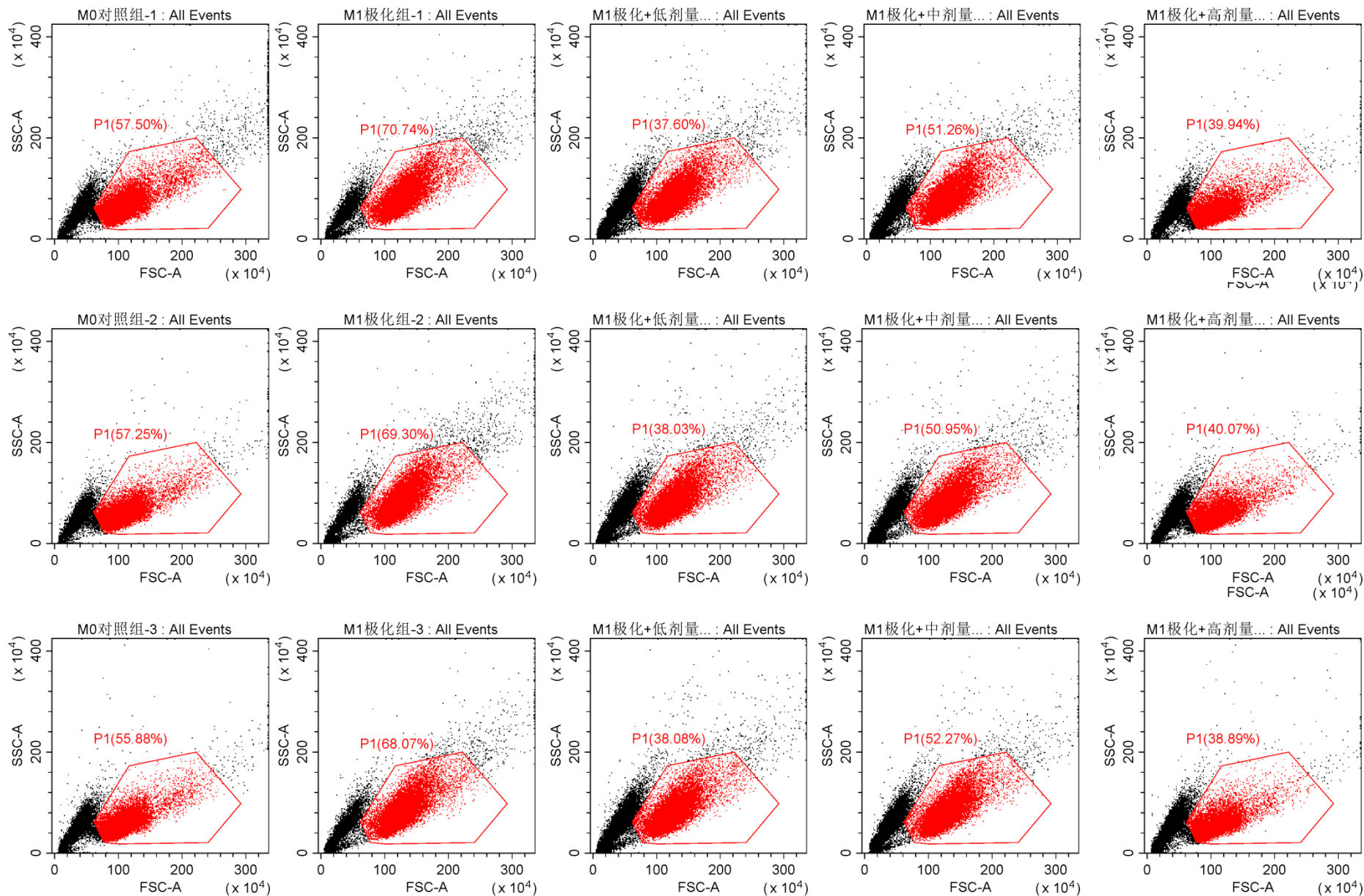

# M1

# M1+TAN-H

# M1+Jagged1

# M1+TAN-H+Jagged1

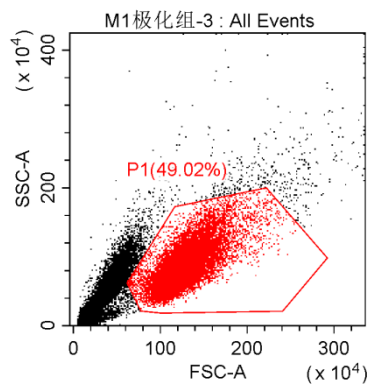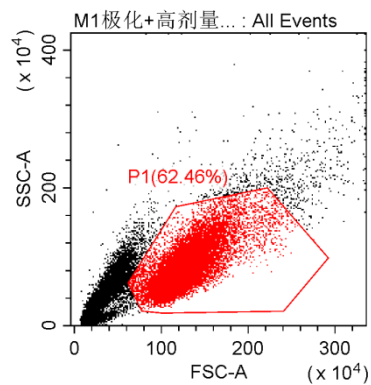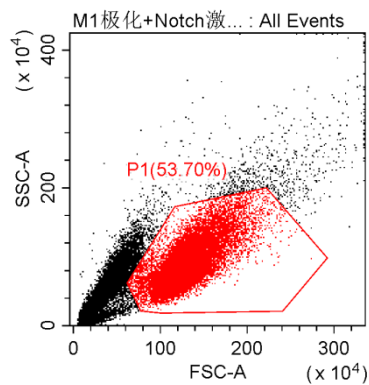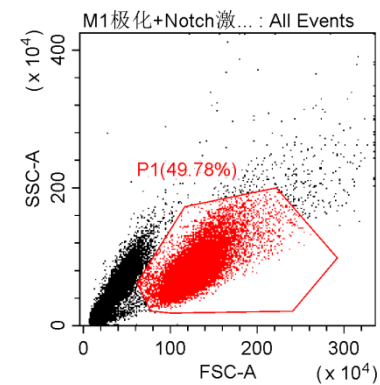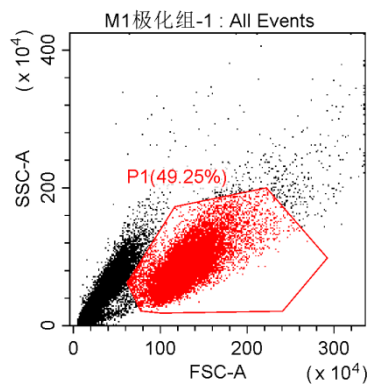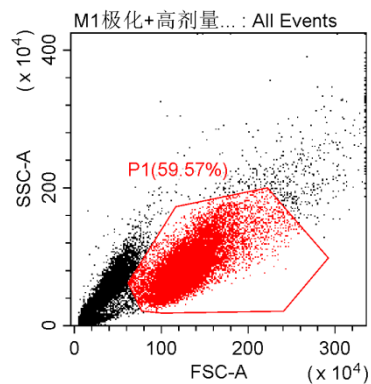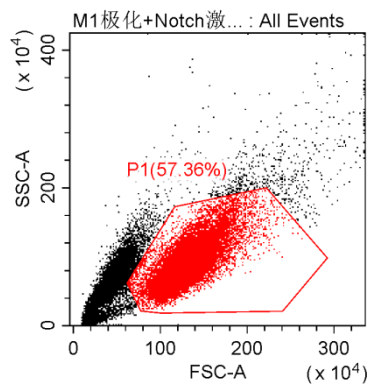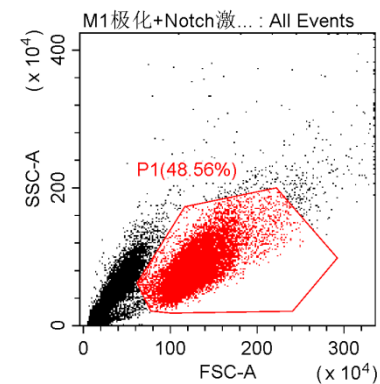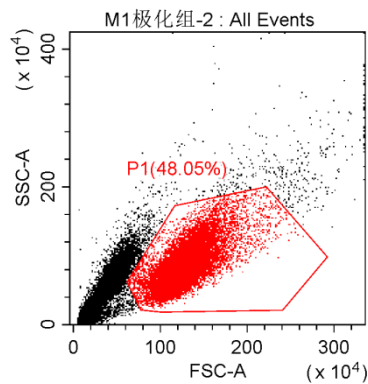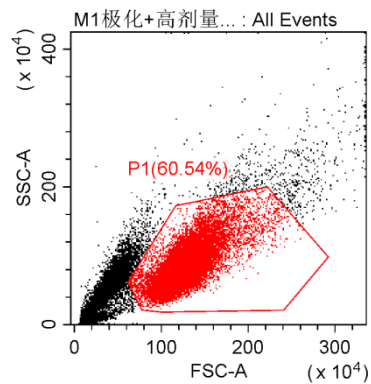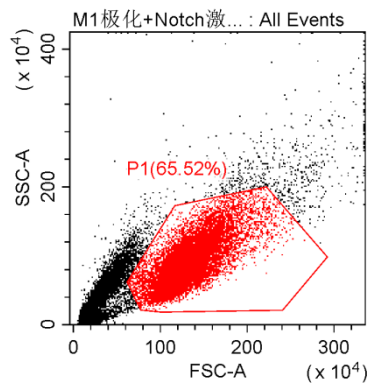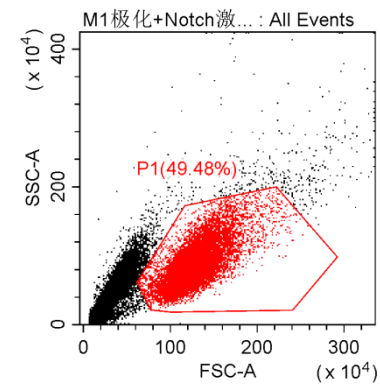

Supplement: Supplementary file 1 — Supplementary Material 1 [file 210_2024_3166_MOESM1_ESM.pdf]
