# Supplementary material for: Tanshinol ameliorates imiquimod-induced psoriasis by inhibiting M1 macrophage polarization through suppression of the notch signaling pathway
Source: Naunyn Schmiedebergs Arch Pharmacol. 2024 Jun 4;397(11):8745–58. doi: 10.1007/s00210-024-03166-9 (PMC11522191; doi:10.1007/s00210-024-03166-9)
Supplement: Supplementary file 2 — Supplementary Material 2 [file 210_2024_3166_MOESM2_ESM.pptx]

## Slide 1
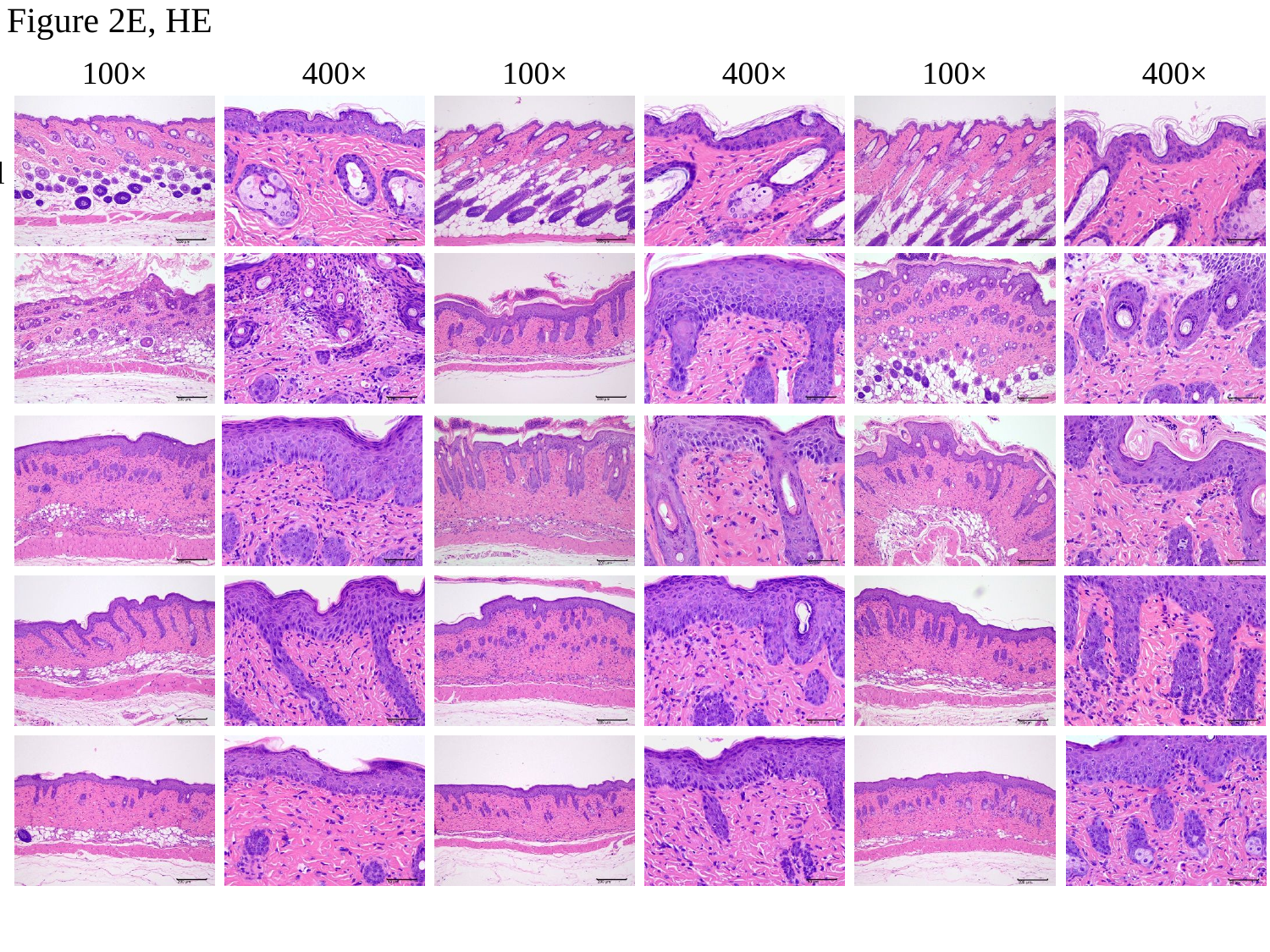

Figure 2E, HE
100×
400×
100×
400×
100×
400×
Control
Model
TAN-L
TAN-M
TAN-H

## Slide 2
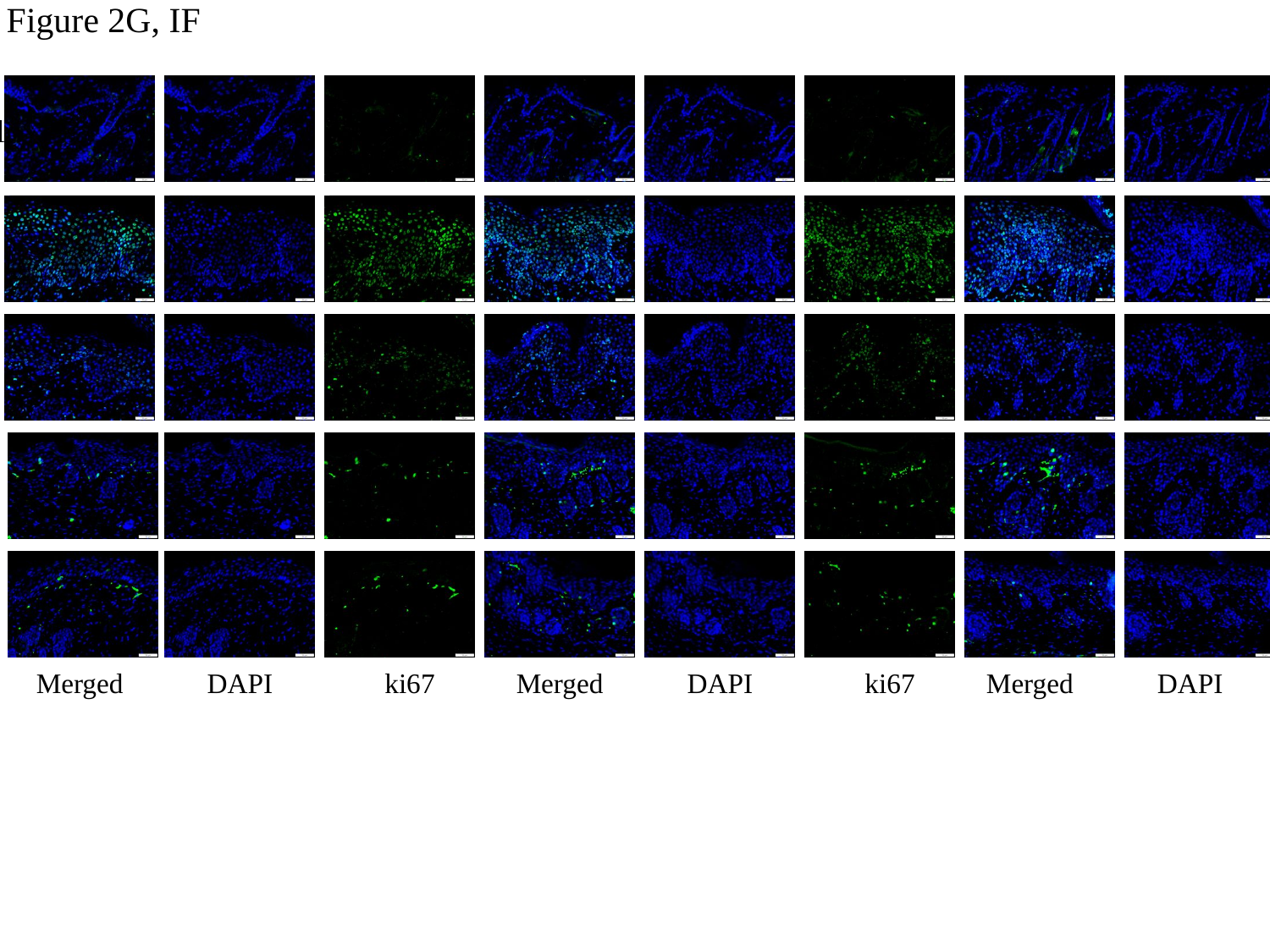

Figure 2G, IF
Control
Model
TAN-L
TAN-M
TAN-H
Merged
DAPI
ki67
Merged
DAPI
ki67
Merged
DAPI
ki67

## Slide 3
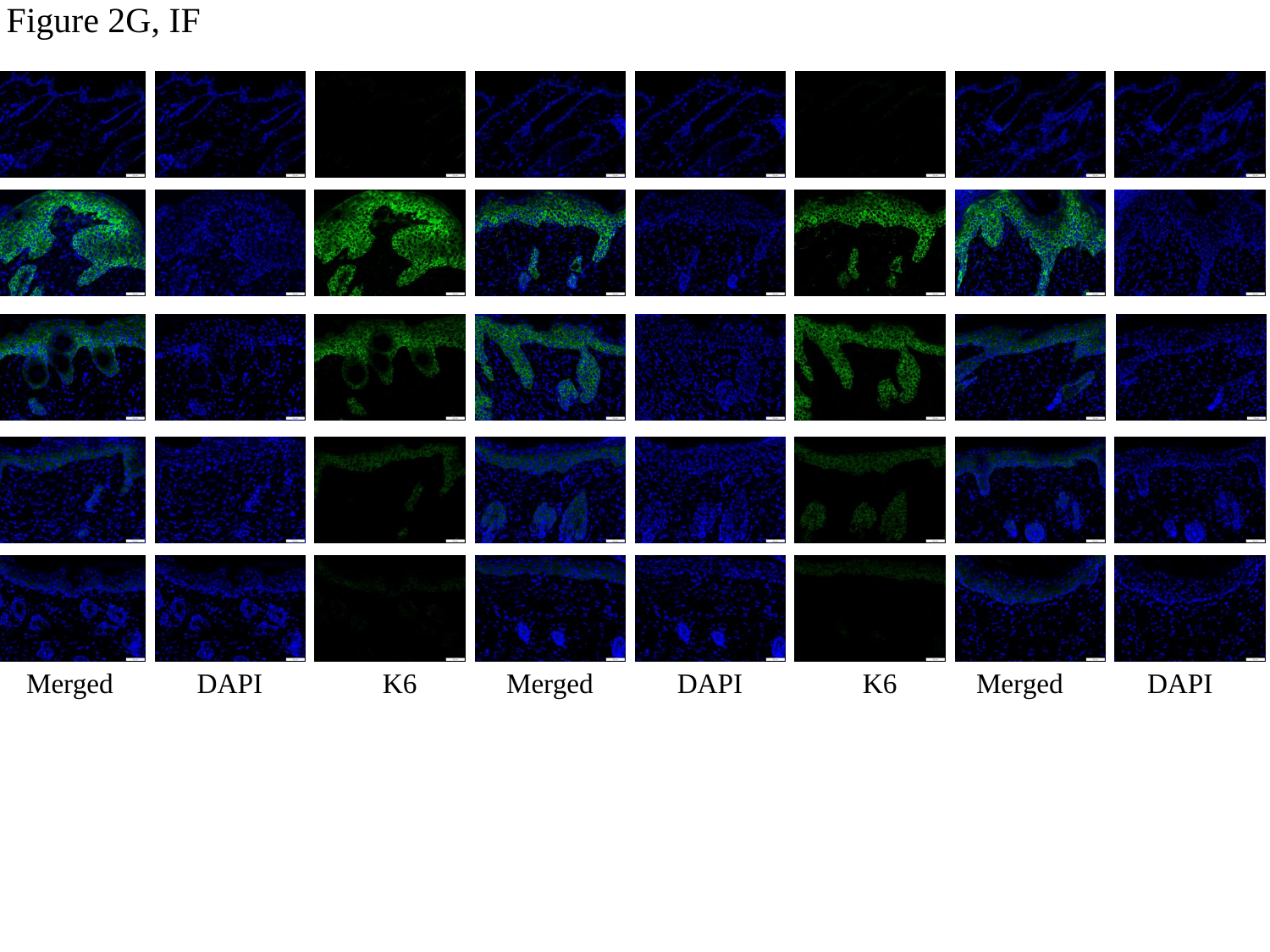

Figure 2G, IF
Control
Model
TAN-L
TAN-M
TAN-H
Merged
DAPI
K6
Merged
DAPI
K6
Merged
DAPI
K6

## Slide 4
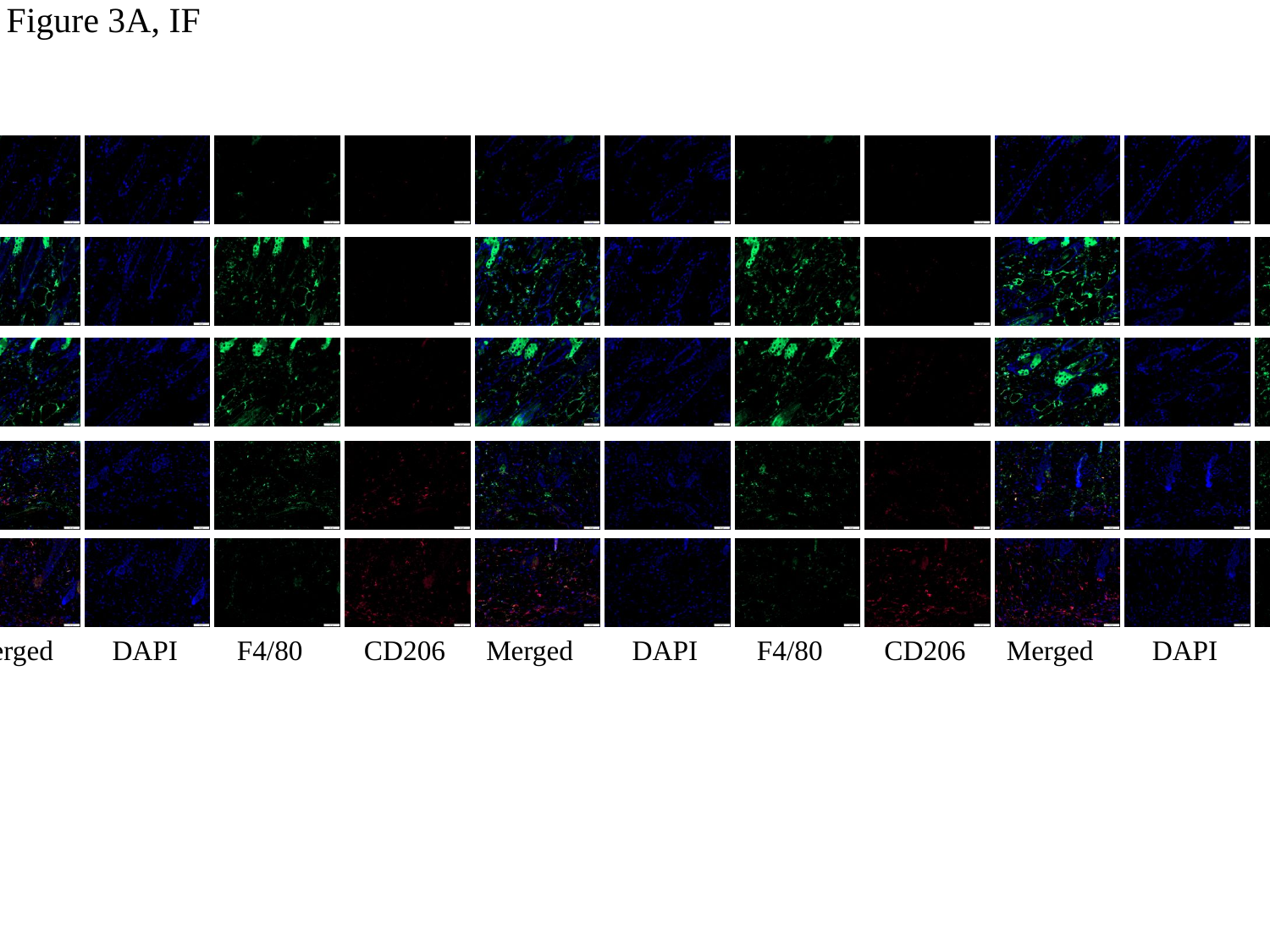

Figure 3A, IF
Control
Model
TAN-L
TAN-M
TAN-H
Merged
DAPI
F4/80
CD206
Merged
DAPI
F4/80
CD206
Merged
DAPI
F4/80
CD206

## Slide 5
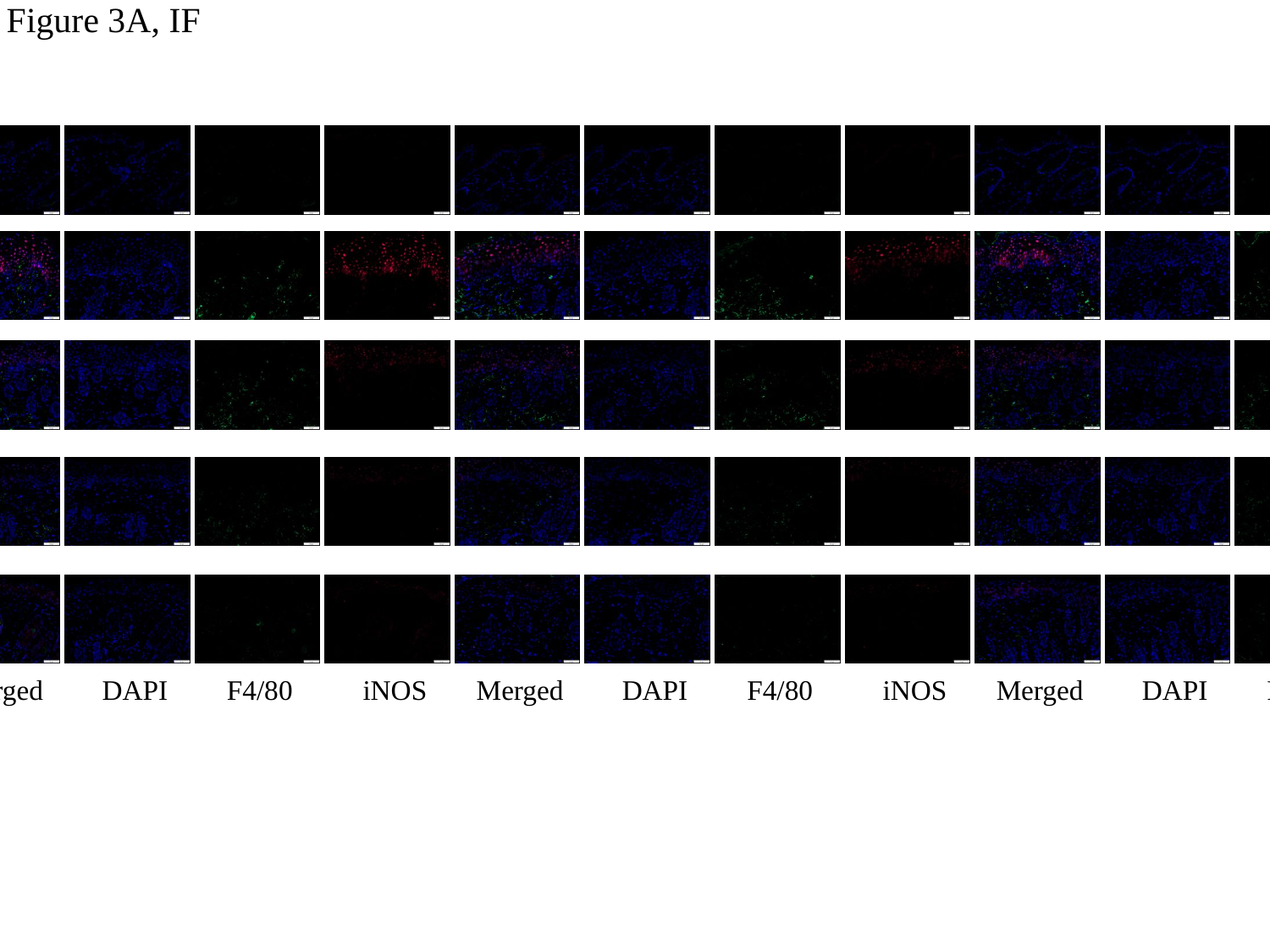

Figure 3A, IF
Control
Model
TAN-L
TAN-M
TAN-H
Merged
DAPI
F4/80
iNOS
Merged
DAPI
F4/80
iNOS
Merged
DAPI
F4/80
iNOS

## Slide 6
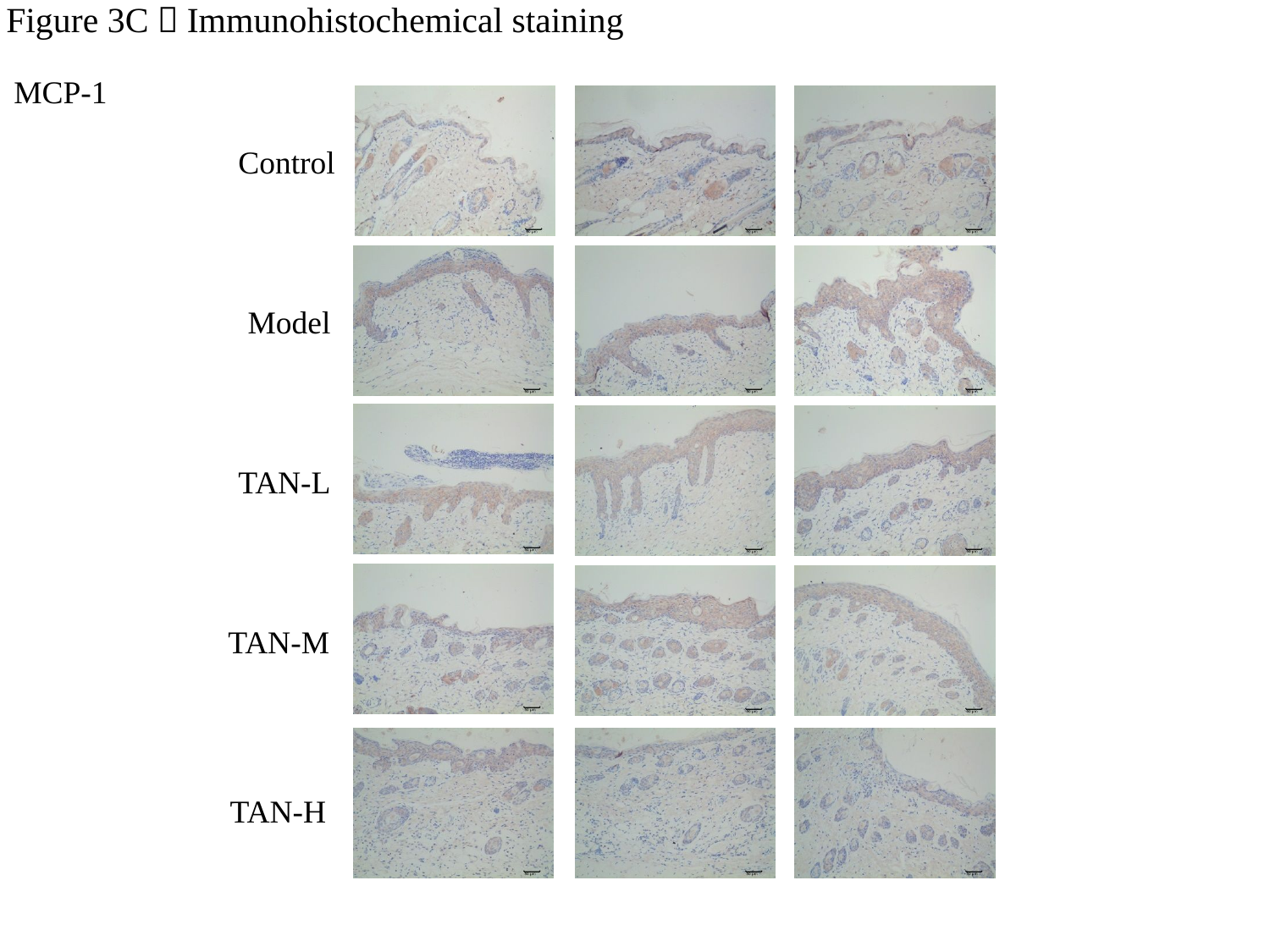

Figure 3C，Immunohistochemical staining
MCP-1
Control
Model
TAN-L
TAN-M
TAN-H

## Slide 7
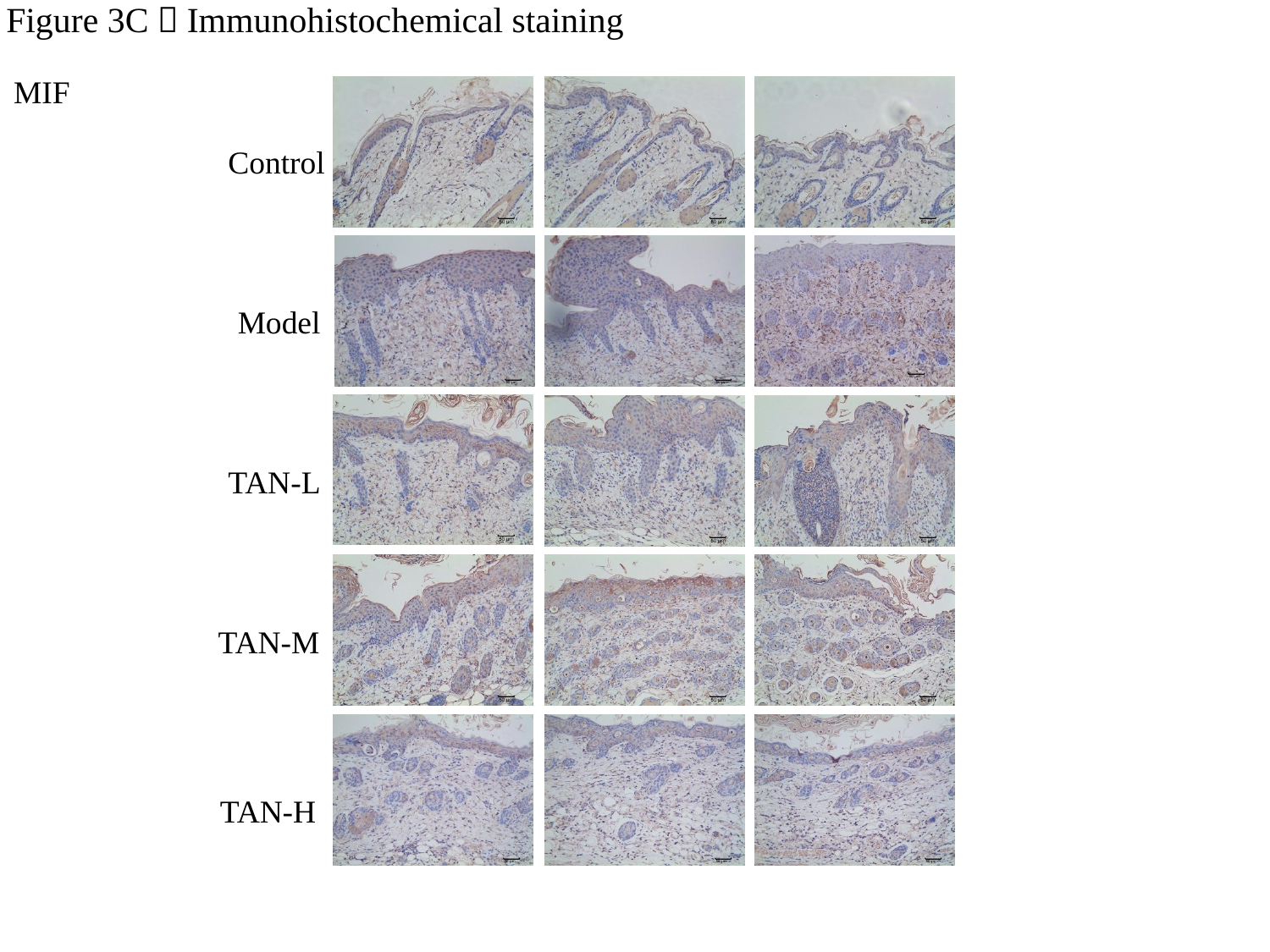

Figure 3C，Immunohistochemical staining
MIF
Control
Model
TAN-L
TAN-M
TAN-H

## Slide 8
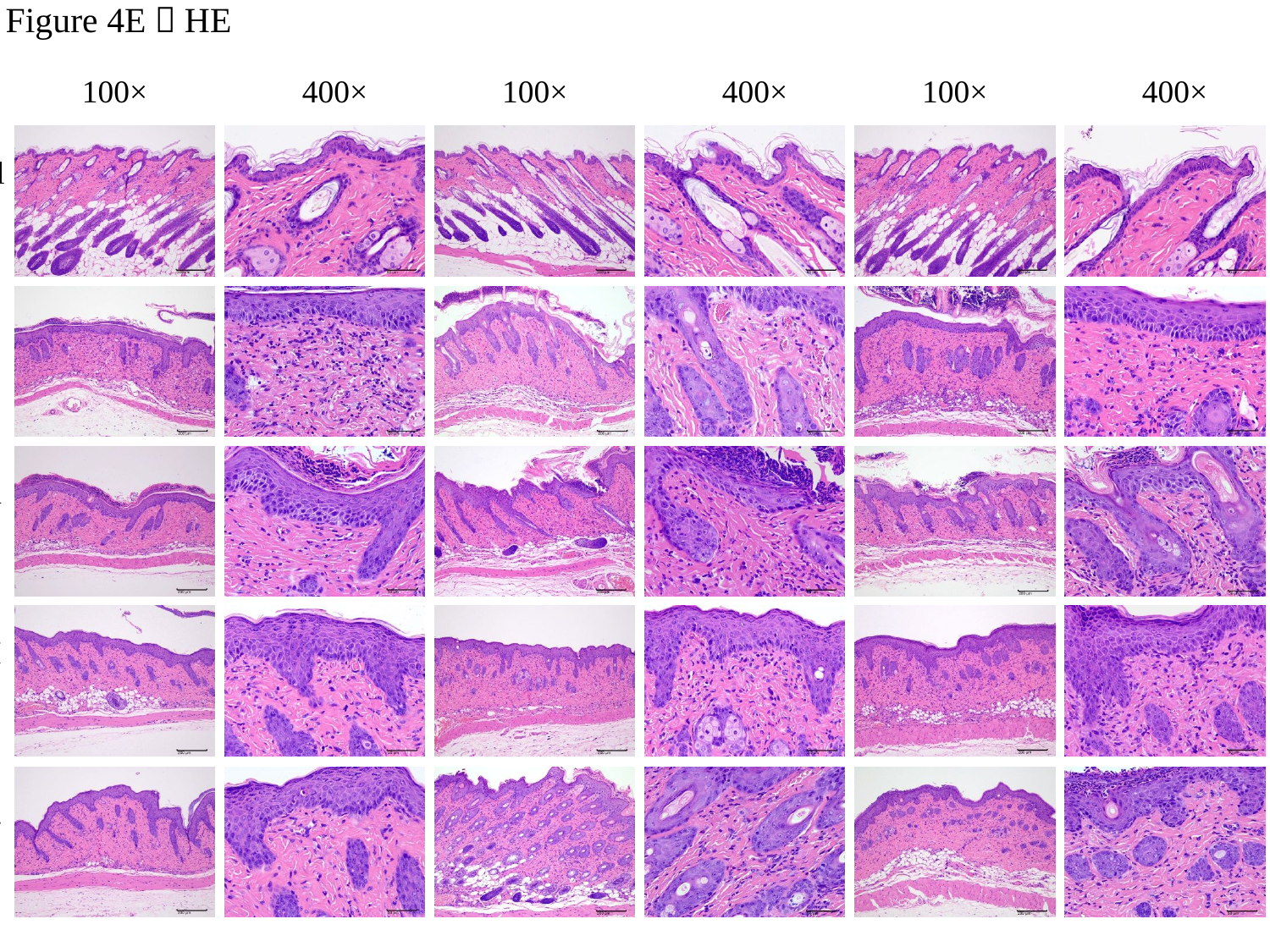

Figure 4E，HE
100×
400×
100×
400×
100×
400×
Control
Model
Clo-A
TAN-H
TAN-H+
Clo-A

## Slide 9
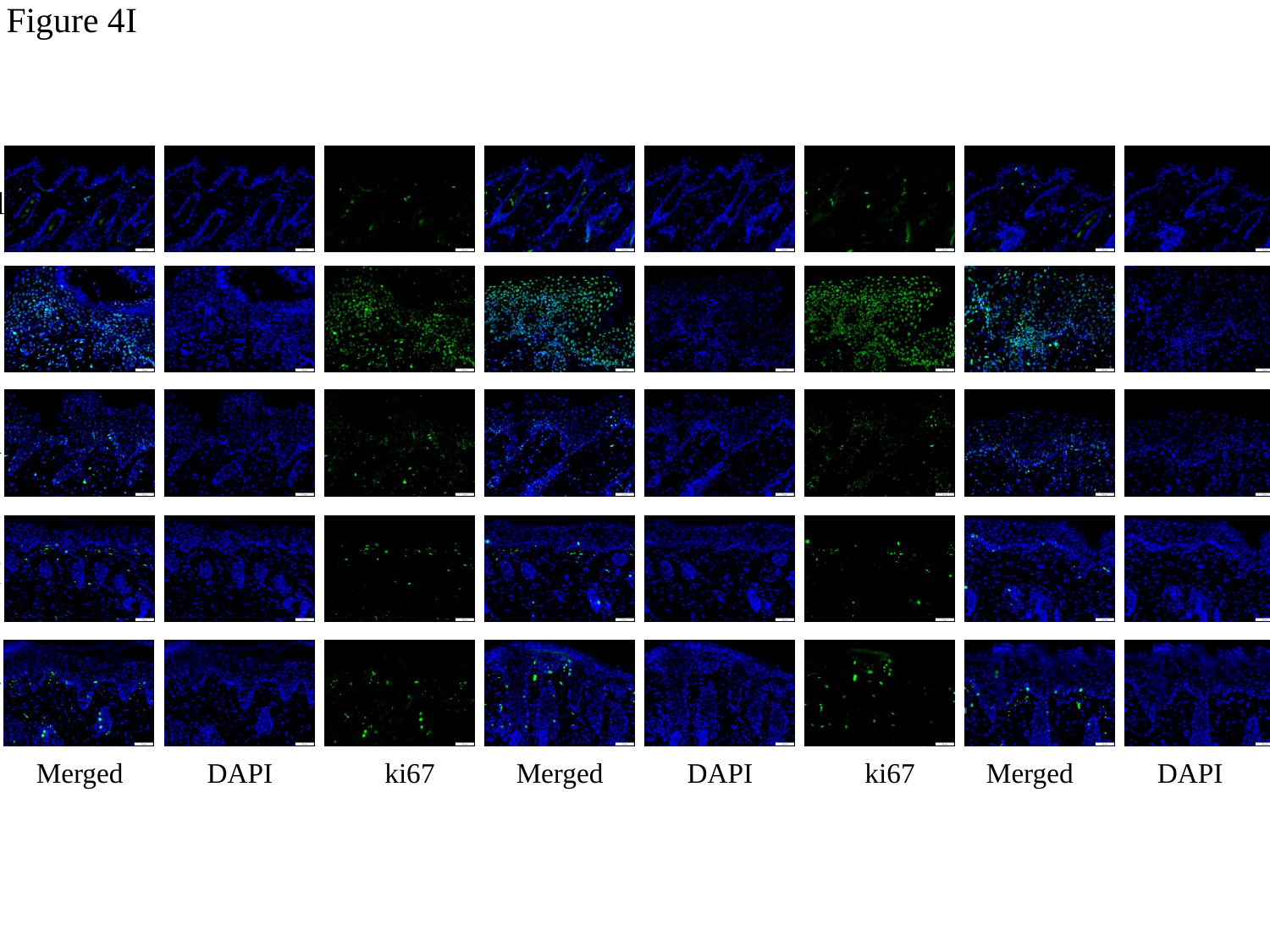

Figure 4I
Control
Model
Clo-A
TAN-H
TAN-H+
Clo-A
Merged
DAPI
ki67
Merged
DAPI
ki67
Merged
DAPI
ki67

## Slide 10
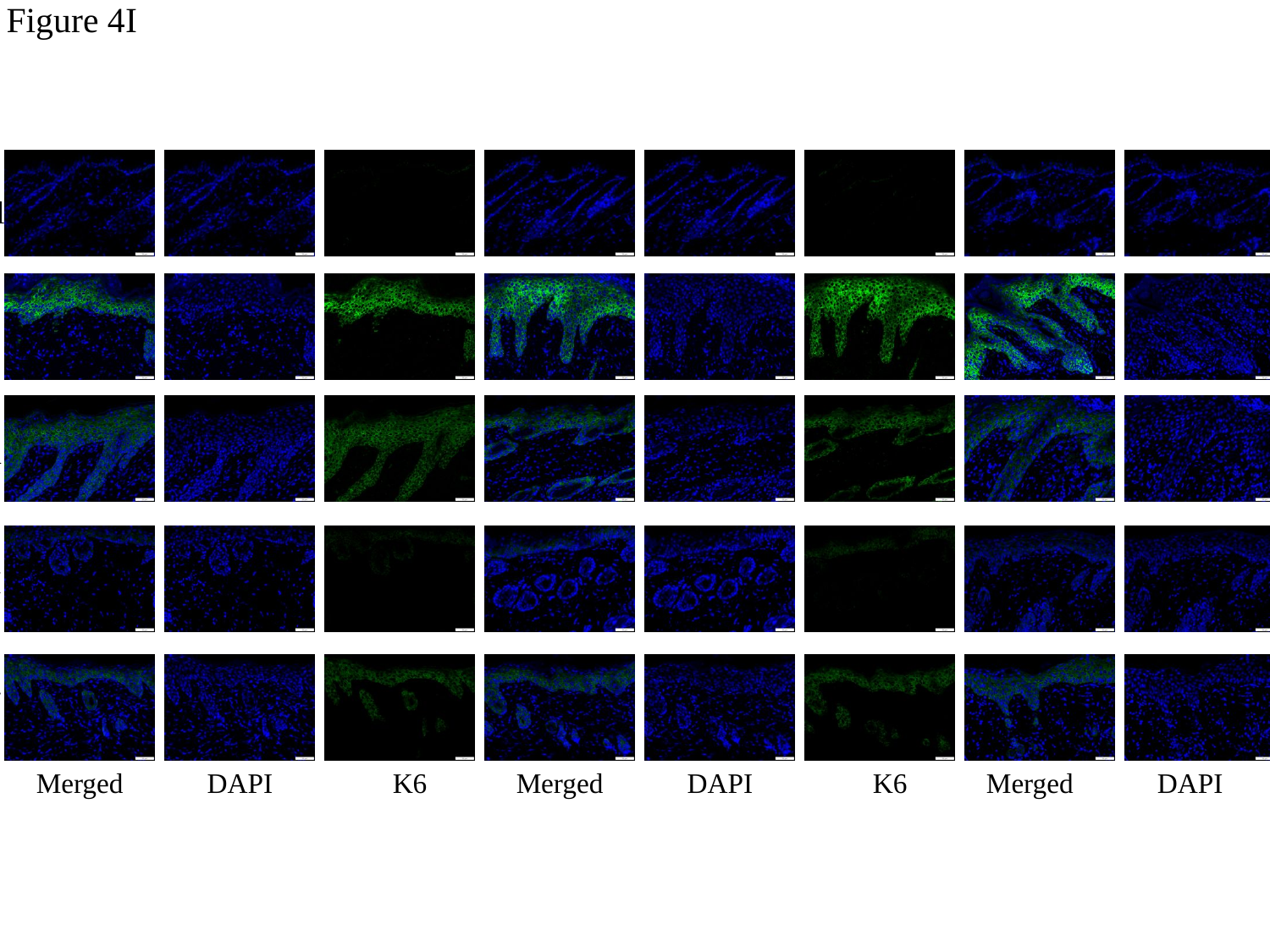

Figure 4I
Control
Model
Clo-A
TAN-H
TAN-H+
Clo-A
Merged
DAPI
K6
Merged
DAPI
K6
Merged
DAPI
K6

## Slide 11
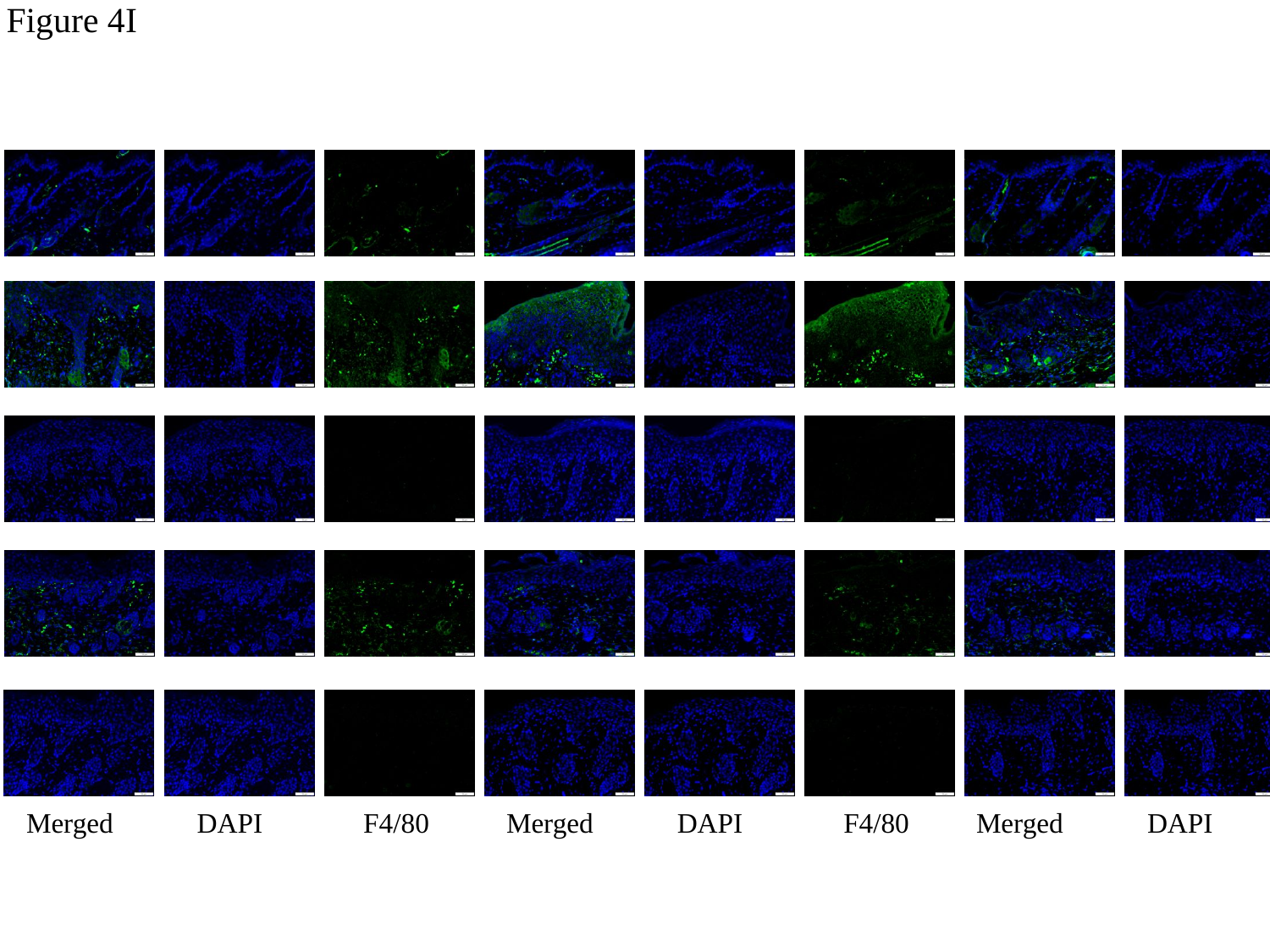

Figure 4I
Control
Model
Clo-A
TAN-H
TAN-H+
Clo-A
Merged
DAPI
F4/80
Merged
DAPI
F4/80
Merged
DAPI
F4/80

## Slide 12
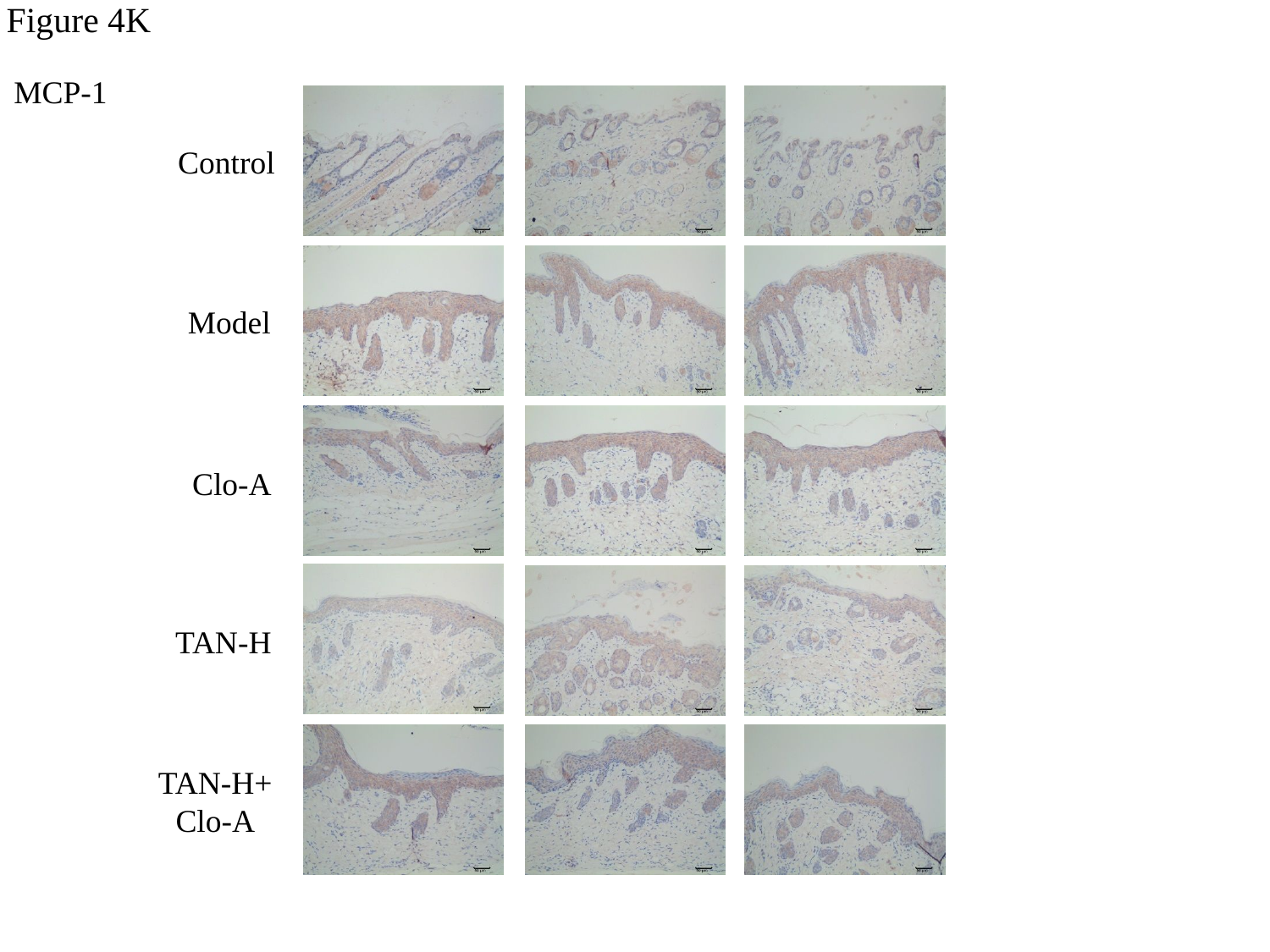

Figure 4K
MCP-1
Control
Model
Clo-A
TAN-H
TAN-H+
Clo-A

## Slide 13
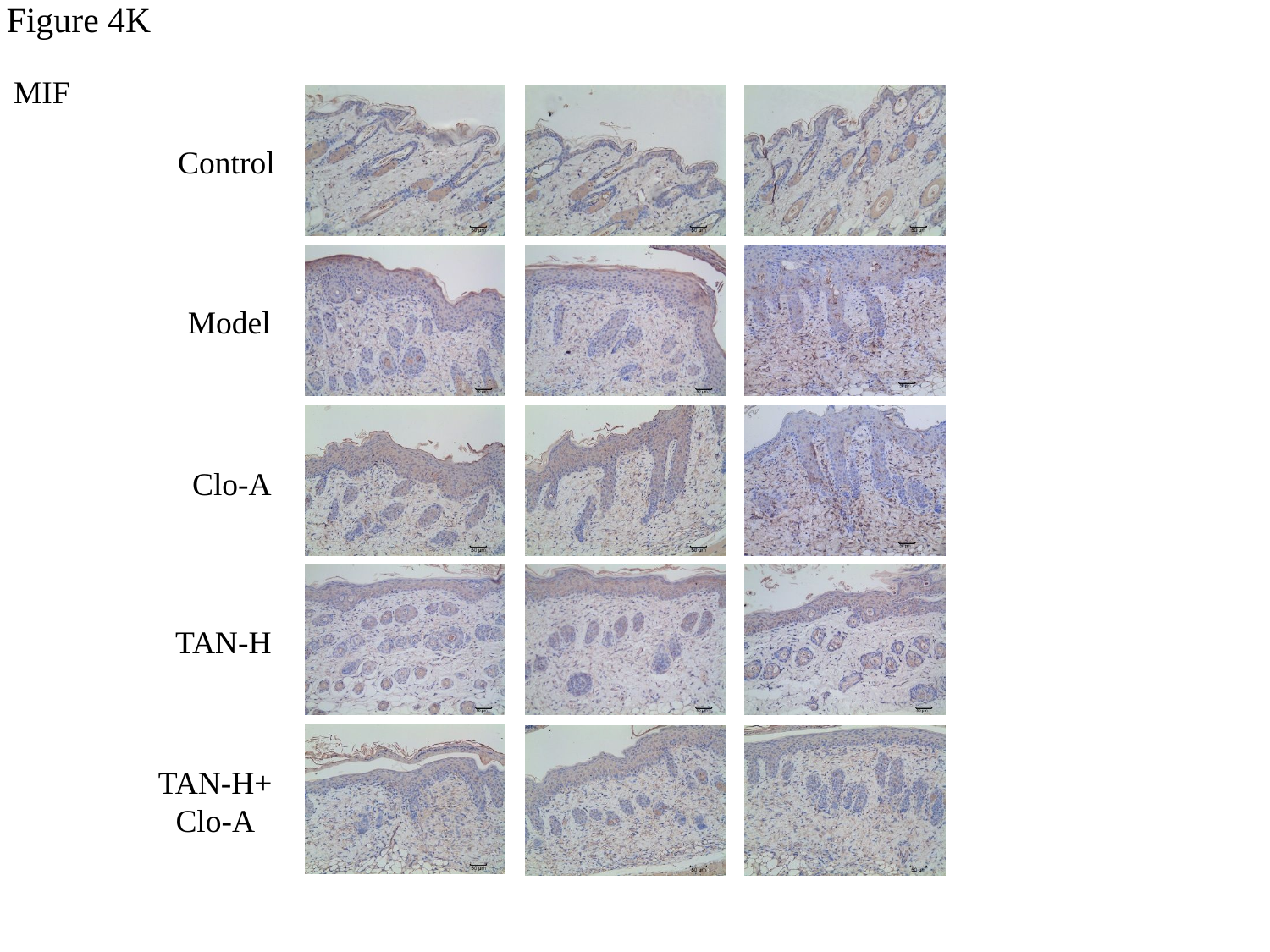

Figure 4K
MIF
Control
Model
Clo-A
TAN-H
TAN-H+
Clo-A

## Slide 14
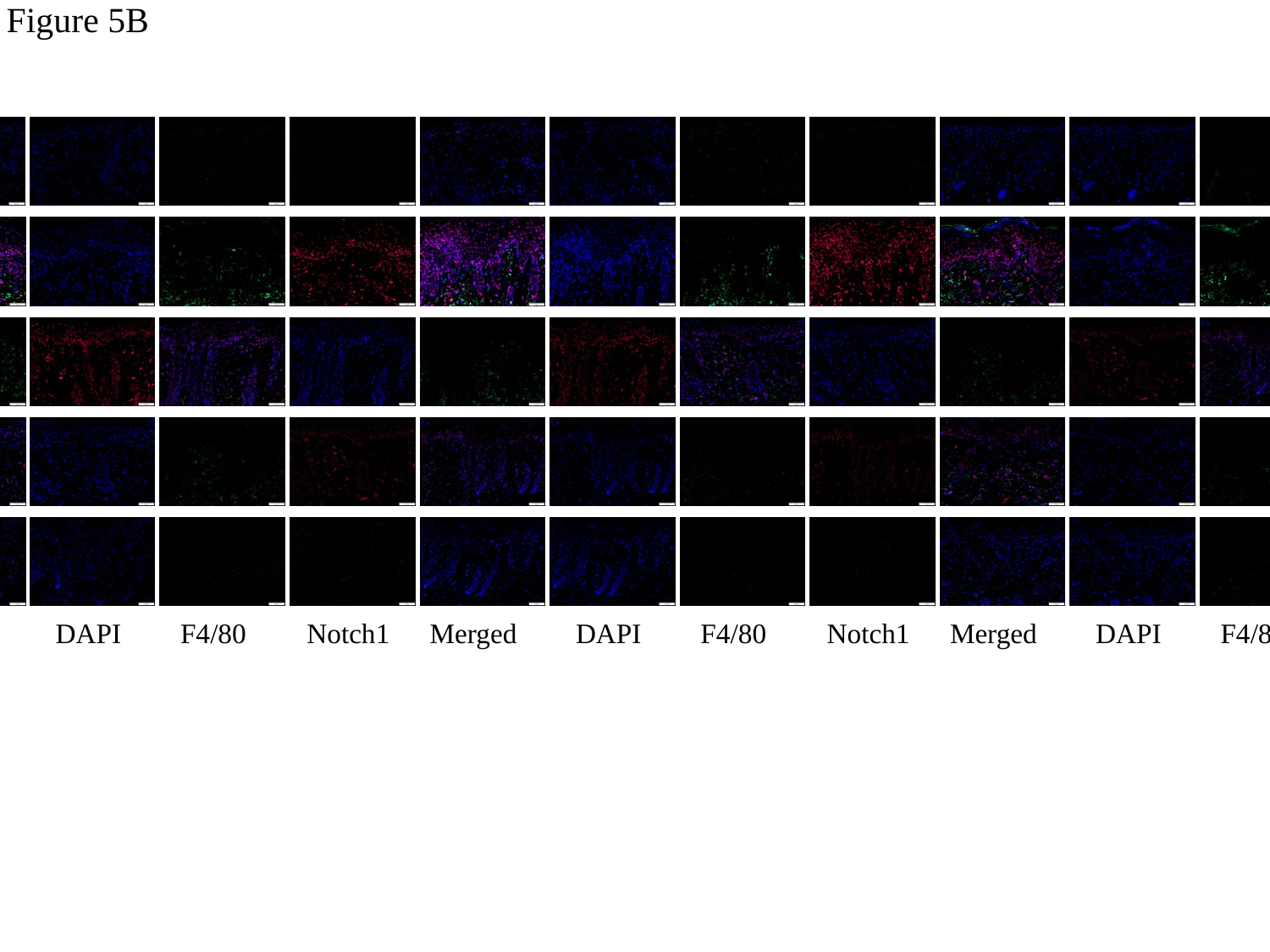

Figure 5B
Control
Model
TAN-L
TAN-M
TAN-H
Merged
DAPI
F4/80
Notch1
Merged
DAPI
F4/80
Notch1
Merged
DAPI
F4/80
Notch1

## Slide 15
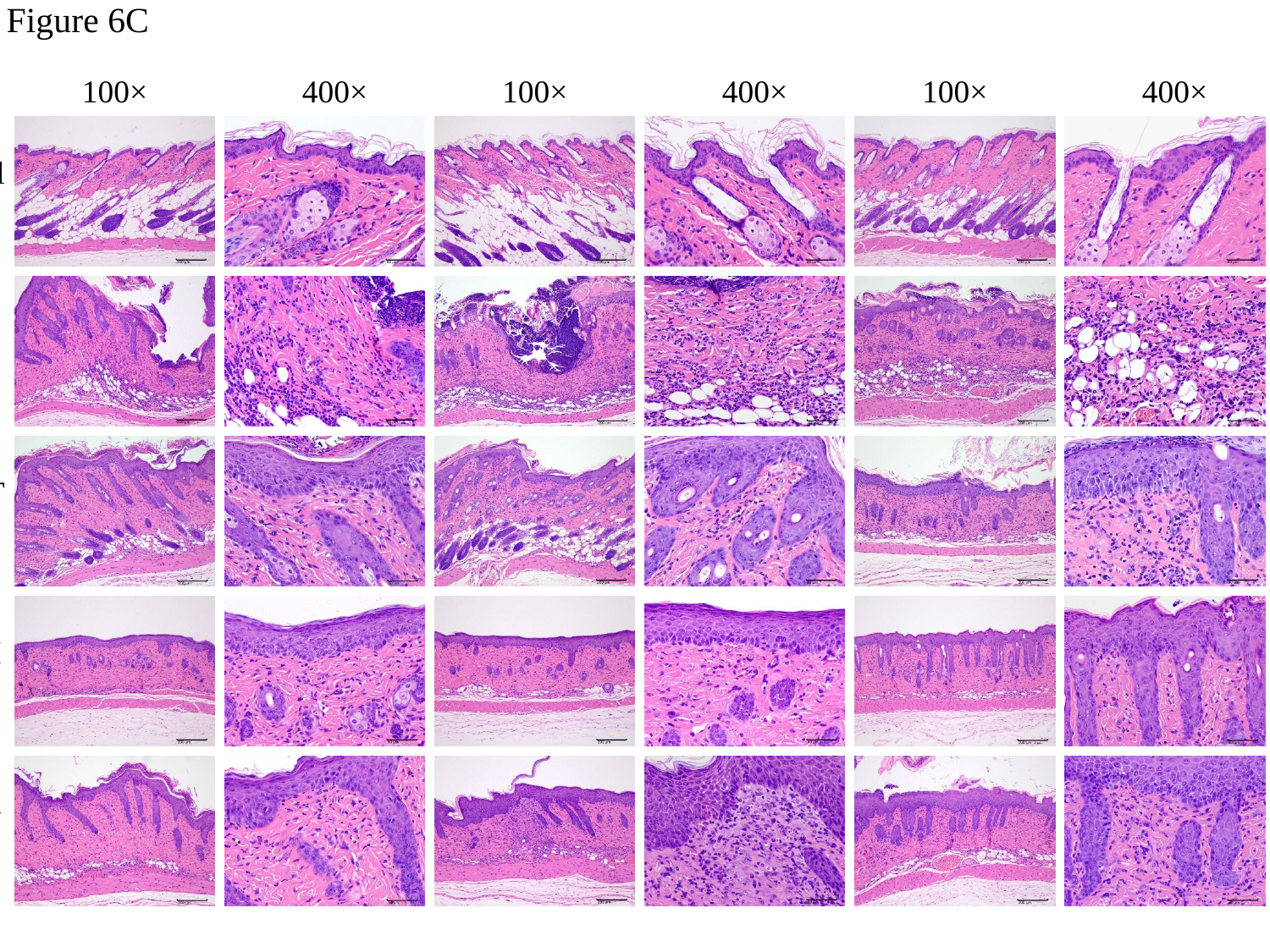

Figure 6C
100×
400×
100×
400×
100×
400×
Control
Model
DAPT
TAN-H
TAN-H+
Jagged1

## Slide 16
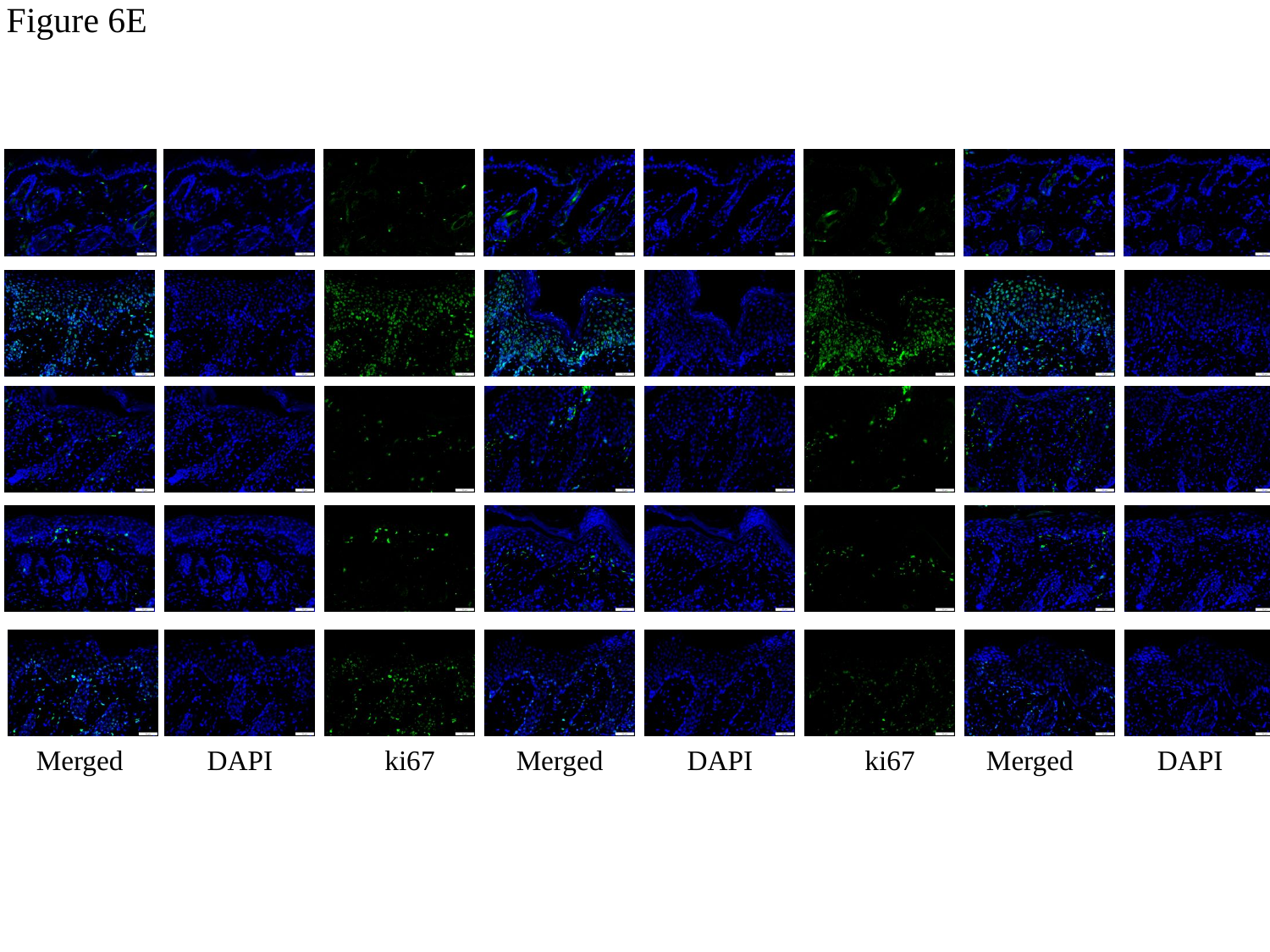

Figure 6E
Control
Model
DAPT
TAN-H
TAN-H+
Jagged1
Merged
DAPI
ki67
Merged
DAPI
ki67
Merged
DAPI
ki67

## Slide 17
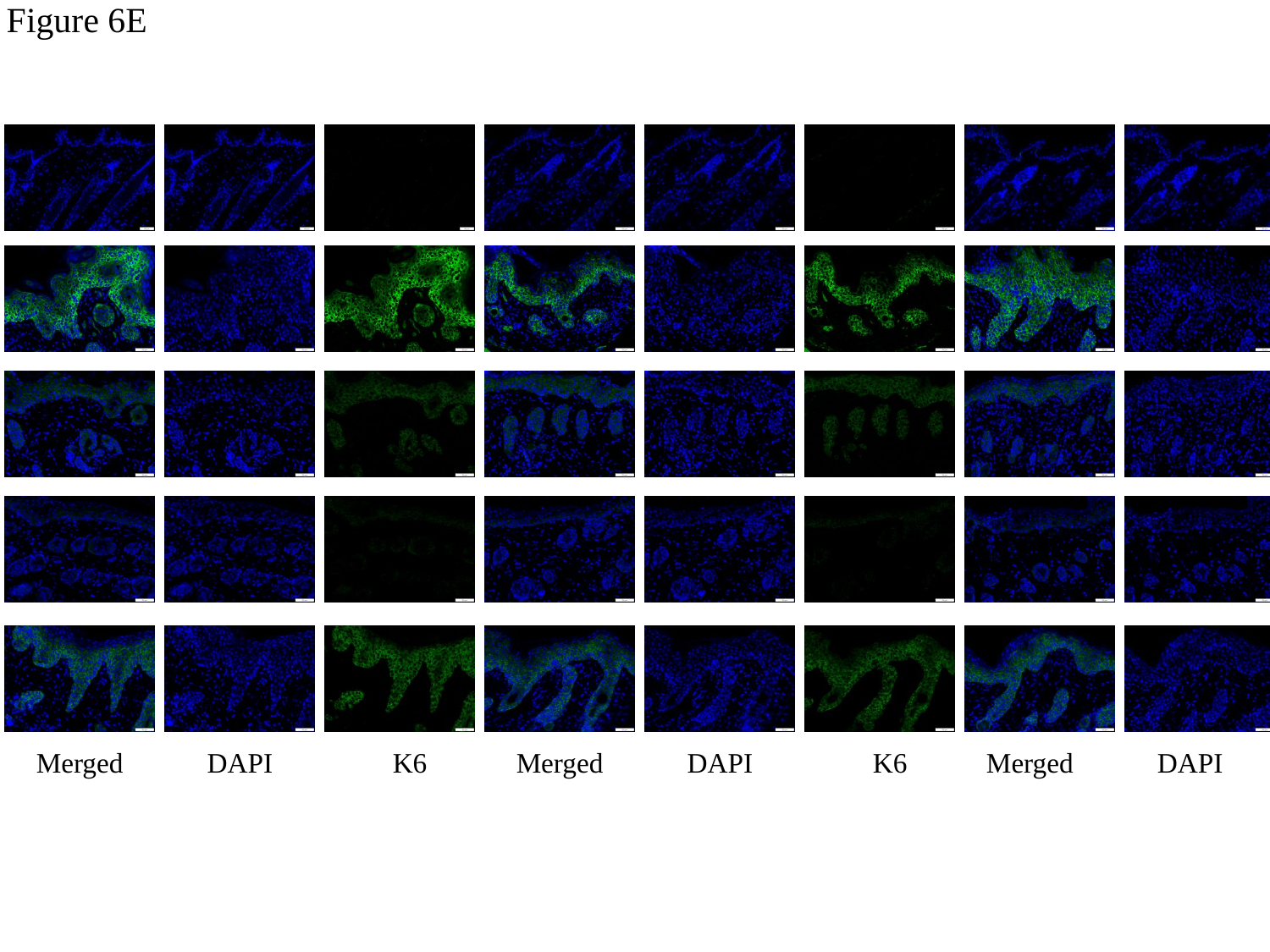

Figure 6E
Control
Model
DAPT
TAN-H
TAN-H+
Jagged1
Merged
DAPI
K6
Merged
DAPI
K6
Merged
DAPI
K6

## Slide 18
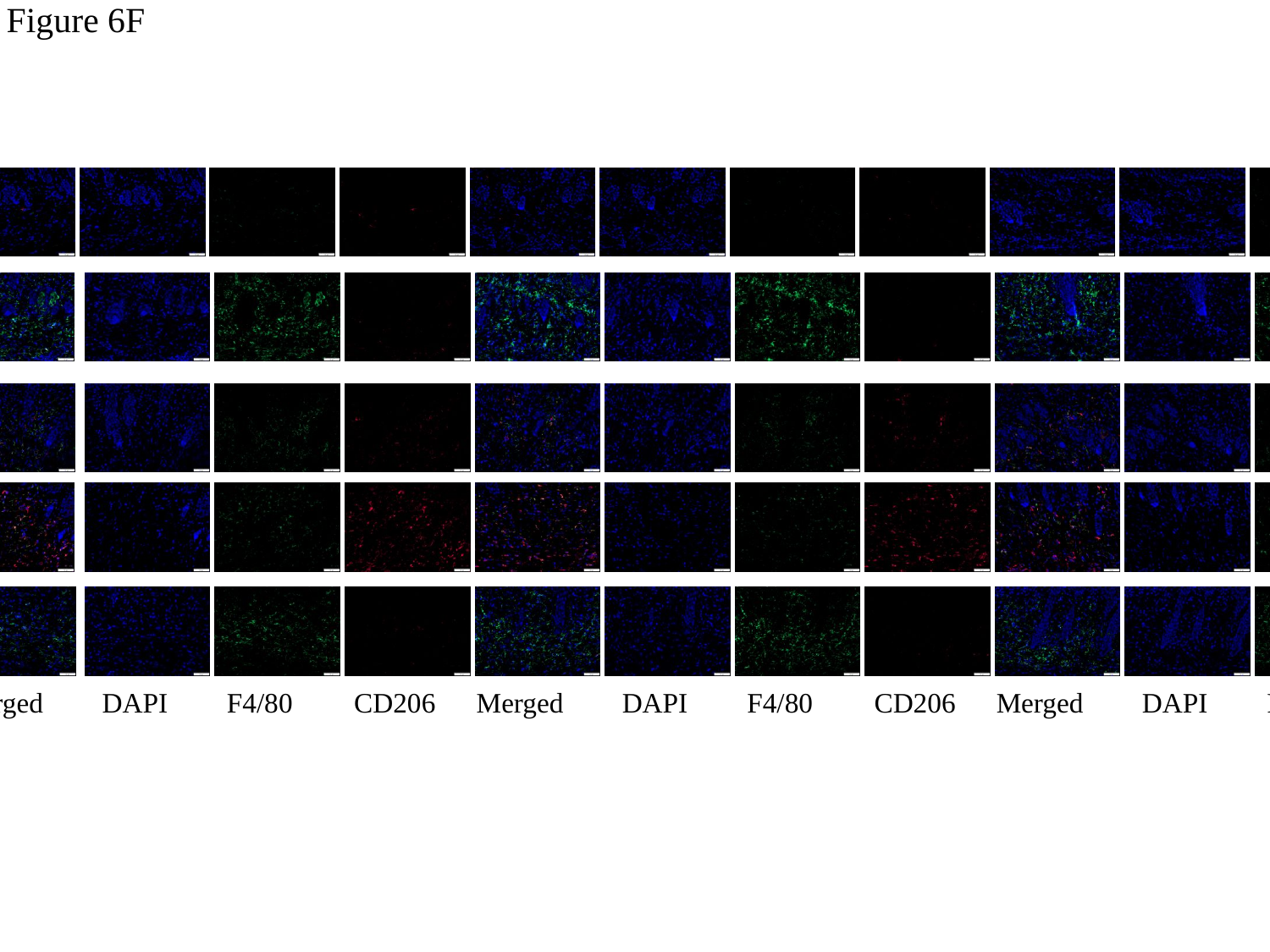

Figure 6F
Control
Model
DAPT
TAN-H
TAN-H+
Jagged1
Merged
DAPI
F4/80
CD206
Merged
DAPI
F4/80
CD206
Merged
DAPI
F4/80
CD206

## Slide 19
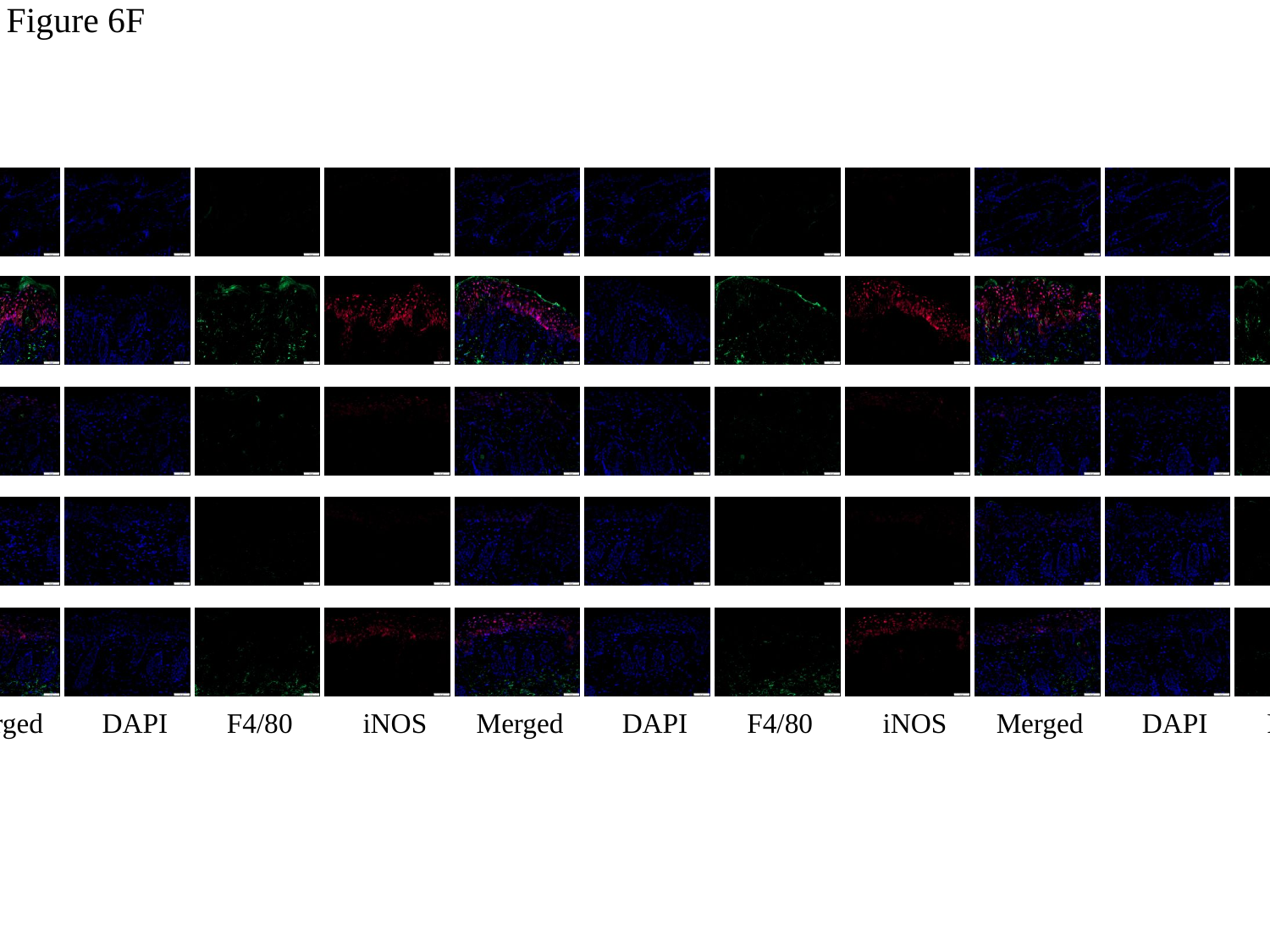

Figure 6F
Control
Model
DAPT
TAN-H
TAN-H+
Jagged1
Merged
DAPI
F4/80
iNOS
Merged
DAPI
F4/80
iNOS
Merged
DAPI
F4/80
iNOS

## Slide 20
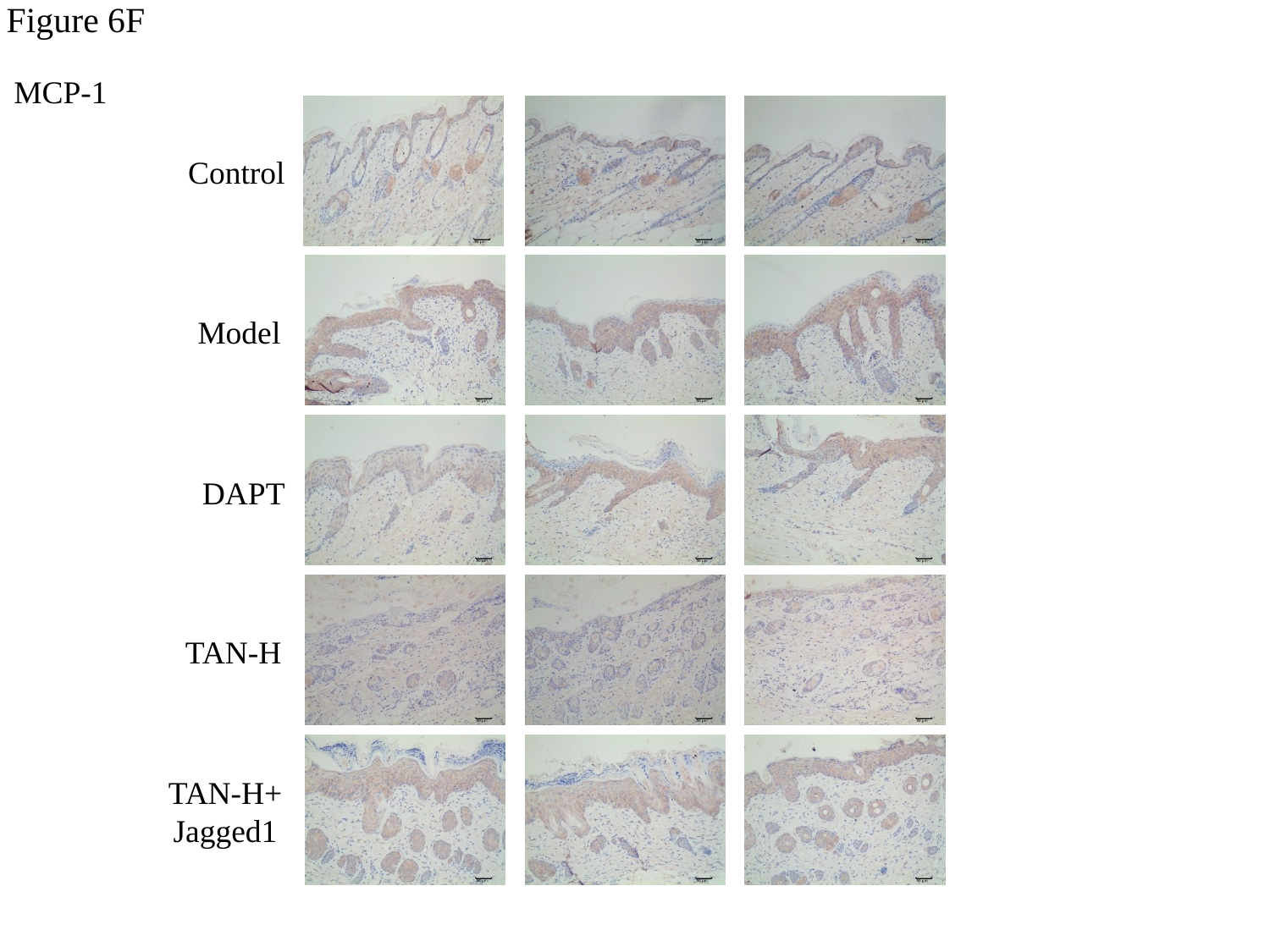

Figure 6F
MCP-1
Control
Model
DAPT
TAN-H
TAN-H+
Jagged1

## Slide 21
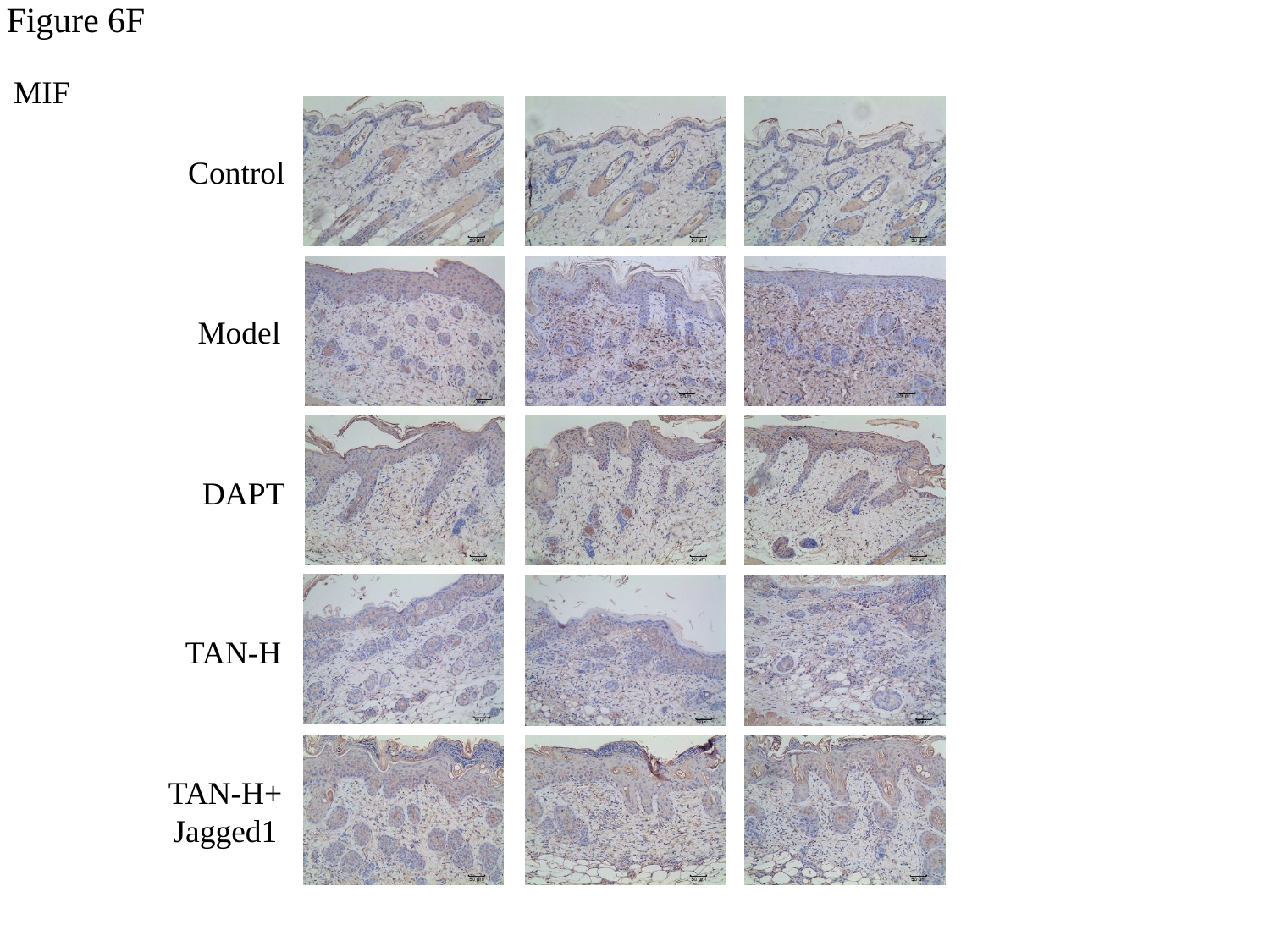

Figure 6F
MIF
Control
Model
DAPT
TAN-H
TAN-H+
Jagged1
