# Supplementary material for: Tanshinol ameliorates imiquimod-induced psoriasis by inhibiting M1 macrophage polarization through suppression of the notch signaling pathway
Source: Naunyn Schmiedebergs Arch Pharmacol. 2024 Jun 4;397(11):8745–58. doi: 10.1007/s00210-024-03166-9 (PMC11522191; doi:10.1007/s00210-024-03166-9)
Supplement: Supplementary file 3 — Supplementary Material 3 [file 210_2024_3166_MOESM3_ESM.docx]

**Figure 5**

Notch1

Hey1

Hes1

**Figure 6**

Notch1

Hey1

Hes1

**Figure 7**

Notch1

Hey1

Hes1

**Figure 8**

Notch1

Hey1

Hes1
